# Suppression of lung cancer progression by isoliquiritigenin through its metabolite 2, 4, 2’, 4’-Tetrahydroxychalcone

**DOI:** 10.1186/s13046-018-0902-4

**Published:** 2018-10-03

**Authors:** Changliang Chen, Anitha K. Shenoy, Ravi Padia, Dongdong Fang, Qing Jing, Ping Yang, Shi-Bing Su, Shuang Huang

**Affiliations:** 1 Research Center for Traditional Chinese Medicine Complexity System, Shanghai University of Traditional Chinese Medicine, Shanghai, China; 2 Department of Anatomy and Cell Biology, University of Florida College of Medicine, Gainesville, FL 32610 USA; 3 Department of Pharmaceutics and Biomedical Sciences, California Health Sciences University, Clovis, CA USA; 4 Department of Cardiology, Changhai Hospital, Shanghai, China; 5 Instrumental Analysis Center, School of Pharmacy, Fudan University, Shanghai, China

**Keywords:** Isoliquitigenin, Src, Cell migration, Lung cancer, Metabolite

## Abstract

**Background:**

Licorice is an herb extensively used for both culinary and medicinal purposes. Various constituents of licorice have been shown to exhibit anti-tumorigenic effect in diverse cancer types. However, majority of these studies focus on the aspect of their growth-suppressive role. In this study, we systematically analyzed known licorice’s constituents on the goal of identifying component(s) that can effectively suppress both cell migration and growth.

**Methods:**

Effect of licorice’s constituents on cell growth was evaluated by MTT assay while cell migration was assessed by both wound-healing and Transwell assays. Cytoskeleton reorganization and focal adhesion assembly were visualized by immunofluorescence staining with labeled phalloidin and anti-paxillin antibody. Activity of Src in cells was judged by western blot using phosphor-Src416 antibody while Src kinase activity was measured using Promega Src kinase assay system. Anti-tumorigenic capabilities of isoliquiritigenin (ISL) and 2, 4, 2′, 4’-Tetrahydroxychalcone (THC) were investigated using lung cancer xenograft model.

**Results:**

Using a panel of lung cancer cell lines, ISL was identified as the only licorice’s constituent capable of inhibiting both cell migration and growth. ISL-led inhibition in cell migration resulted from impaired cytoskeleton reorganization and focal adhesion assembly. Assessing the phosphorylation of 141 cytoskeleton dynamics-associated proteins revealed that ISL reduced the abundance of Tyr421-phosphorylation of cortactin, Tyr925- and Tyr861-phosphorylation of FAK, indicating the involvement of Src because these sites are known to be phosphorylated by Src. Enigmatically, ISL inhibited Src in cells while displayed no effect on Src activity in cell-free system. The discrepancy was explained by the observation that THC, one of the major ISL metabolite identified in lung cancer cells abrogated Src activity both in cells and cell-free system. Similar to ISL, THC deterred cell migration and abolished cytoskeleton reorganization/focal adhesion assembly. Furthermore, we showed both ISL and THC suppressed in vitro lung cancer cell invasion and in vivo tumor progression.

**Conclusion:**

Our study suggests that ISL inhibits lung cancer cell migration and tumorigenesis by interfering with Src through its metabolite THC. As licorice is safely used for culinary purposes, our study suggests that ISL or THC may be safely used as a Src inhibitor.

**Electronic supplementary material:**

The online version of this article (10.1186/s13046-018-0902-4) contains supplementary material, which is available to authorized users.

## Background

Radix Glycyrrhiza (Licorice) has been used to treat cancer in China for thousands of years. Licorice contains various types of flavonoids and triterpenes, and many of them have been found to possess anti-tumorigenic activities. Isoliquiritigenin (ISL), one of the flavonoids in licorice, can inhibit growth of diverse cancer types including breast, lung, cervical, ovary, prostate and colon cancer by inducing apoptosis, autophagy and causing DNA damage [1–6]. ISL has also been shown to exhibit inhibitory effect on migration of breast and prostate cancer cells [7–9]. Recently, ISL has been shown to potently suppress imflammatory responses by blocking Toll-like receptor 4 signaling and inflammasome activation [10, 11]. As tumor-promoting inflammation is recognized as a hallmark of cancer [12], ISL may also elicit its tumor-suppressive effect through immune regulation. In xenograft models, ISL was reported to effectively suppress breast and lung tumorigenesis [13, 14]. Although anti-tumorigenic effect of ISL has been associated with the interference of various signaling pathways including JNK/AP1, PI3K/Akt and VEGF/VEGF2 [8, 14, 15], direct target of ISL remains to be characterized.

Src is member of Src family kinase (SFK) and can be activated through the engagement of diverse cellular receptor types such as integrins, growth factor receptors and G protein-coupled receptors [16]. It is critically involved in a wide spectrum cellular events including cell migration and cell growth. For example, Src phosphorylates cortactin and focal adhesion kinase (FAK) to facilitate cytoskeletal reorganization and focal adhesion turnover, two key steps in cell migration [17–19]. Another example is that Src phosphorylates Yes-associated protein 1, leading to the activation of Hippo signaling pathway and subsequent hyperproliferation in skin squamous carcinoma cells [20]. Numerous studies have convincingly demonstrated the importance of Src in tumor progression and metastasis [21]. For instance, Src often interacts with EGFR and mediate EGFR-associated events [22]. Since oncogenic mutations and aberrant activation of EGFR occurs frequently in various tumor types including non-small cell lung carcinomas (NSCLC) [23, 24], SFK inhibitors are currently used to treat various malignancies [25, 26].

With the aid of 3 well-migratory lung cancer cell lines, we compared various components in licorice for their effect on cell migration. Among all tested components, we showed that ISL was the only compound effectively inhibiting cell migration and its motility-inhibitory effect was through its ability to impair cytoskeleton reorganization and focal adhesion assembly. To elucidate mechanism underlying ISL action, we showed that ISL reduced cortactin’s Tyr421-phosphorylation, FAK’s Tyr861- and Tyr925-phosphorylation, indicating that ISL interfered with Src function because they are known to be Src phosphorylation sites [17, 19, 27, 28]. Intriguingly, ISL was unable to inhibit Src activity in cell-free kinase assay but it decreased the amount of Tyr416-phosphorylated Src in lung cancer cells at a concentration as low as 3 μM. This observation raised the possibility that ISL inhibits Src through its cellular metabolites. In fact, 2, 4, 2′, 4′-tetrahydroxychalcone (THC), one of two main metabolites from ISL (naringenin chalcone was the other) not only decreased Tyr416 phosphorylation of Src in lung cancer cells but also inhibited Src activity in cell-free kinase assay. Moreover, THC suppressed cell migration as well as cytoskeleton reorganization/focal adhesion assembly in similar extent of ISL and specific Src inhibitor PP1. Importantly, both ISL and THC subdued tumor outgrowth and metastasis of H1299 lung cancer cells. In conclusion, this study uncovers that ISL exerts its anti-tumorigenic effect by targeting Src signaling pathway through its metabolite THC.

## Methods

### Cells and other reagents

All cell lines were obtained from America Tissue Culture Collection and their authenticities were confirmed by University of Florida Genomic Core Facility. Cells were maintained in DMEM containing 10% fetal calf serum (FCS) at 37 °C in a humidified incubator supplied with 5% CO_2_. THC was synthesized by Melone Pharmaceutical Co (Dalian, Liaoning, China). All other chemicals and inhibitors were purchased from SigmaAldrich (Saint Louis, MO). Detailed information on antibodies are included in Additional file 1: Table S1.

### Cell growth assay

Cell growth was analyzed by 3-(4, 5-dimethylthiazol-2-yl)-2, 5-diphenyltetrazolium bromide (MTT) assay as previously described [29]. Briefly, 5 × 10^3^ cells were seeded into each well of 24-well culture plates for overnight followed by treatment of individual component of licorice for 2 days. MTT was added to cells for 2 h, MTT formazan crystals were solubilized with DMSO and read at 560 nm on a microplate reader.

### Cell migration and in vitro invasion assays

Cell migration was determined either by Transwell or wound-healing assay as previously described [30–32]. For Transwell assay, the undersurface of Transwells (8.0 mm pore size) was coated with 10 μg/mL of collagen I and serum-free medium was added to lower chambers. Cells were first treated with each component of licorice or Src inhibitor for 24 h, then added to Transwells (10^5^cells in 100 μl/well) and allowed to migrate for 6 h (countable number of cells were present on the undersurface of Transwell at this time point). Cells in upper chamber were removed with cotton swabs, and cells attached on undersurface were stained with crystal violet solution for visualization and counting. For wound-healing assay, cells were grown to confluency and a scratch was generated with a thin pipet tip. Cells were washed with serum-free medium to remove dislodged cells and then incubated in medium in the absence or presence of licorice constituents, PP1, THC or naringenin chalcone for 24 h (Gaps were usually filled by approximately 95% at this time point in untreated cells). Cells were visualized before treatment and at 24 h of incubation period under a phase-contrast microscope. In vitro cell invasion was assessed by Matrigel invasion chambers (Cell Biolabs, San Diego, CA) as previously described [32]. Briefly, 2 × 10^5^ cells were plated into each of invasion chamber and medium containing 10% FCS was added into lower chamber. Cells were allowed to invade for 24 h followed by removal of cells in the invasion chambers by cotton swab. Cells on the undersurface of invasion chambers were stained with crystal violet and counted under a phase-contrast microscope.

### Immunofluorescence staining

Immunofluorescence staining was performed as previously described [33, 34]. Briefly, cells seeded on coverslips were treated with vehicle, 10 μM ISL, PP1 or THC for 24 h followed 1 h stimulation of 10% FCS. Cells were fixed with 4% paraformaldehyde and then permeabilized with 0.5% Triton-×100 followed by incubation with FITC-labeled anti-Paxillin monoclonal antibody and rhodamine-conjugated phalloidin for 1 h. Nuclei were visualized by staining with 4′, 6-diamidino-2-phenylindole (DAPI). The fluorescence staining was observed under a fluorescence microscope (Axiovert 200 M; Carl Zeiss, Thornwood, NY).

### Phospho-protein profiling, western blotting and Src kinase assay

H1299 cells were treated with 3 μM ISL for 24 h and cell lysates were subjected to phosphor-protein profiling analysis using Cytoskeleton Phospho-antibody microarray (Full Moon Biosystems, Sunnyvale, CA) which contains 141 site-specific phopsho-antibodies. For each phospho-specific antibody, a phosphorylation signal ratio was calculated. To analyze how ISL, THC, naringenin chalcone and PP1 affected the levels of phosphorylated cortactin, FAK and Src, overnight-cultured cells were treated with varying concentrations of these compounds for 24 h and then lyzed for western blotting with the respective antibodies as previously described [34]. The effect of ISL, THC, naringenin chalcone and PP1 on Src activity was analyzed using ADP-Glo™ Src kinase assay kit (Promega Corporation, Madison, WI) according to manufacturer’s protocol.

### UHPLC-DAD/Q-TOF mass spectrum

Compounds were identified on UHPLC–DAD/Q-TOF MS system, consisting of an AB-Sciex Triple TOFTM 5600+ mass spectrometer (Sciex, Framingham, MA) couple with an Agilent 1290 Infinity Series UHPLC system (Agilent Technologies, Santa Clara, CA). Chromatographic separation was performed on Acquity UPLC HSS T3 (C18, 1.8 μm, 100 mm × 2.1 mm, Waters) column at 35 °C. The mobile phase consisted of water with 0.1% formic acid (A) and methanol (B). Separation was performed by gradient elution as follows: 20–70% B from 0 to 25 min, 70–95% B from 25 to 30 min, 95–95% B from 30 to 31 min, 95–20% B from 31 to 31.10 min, 20–20% B from 31.10 to 34 min. Injection volume of the test sample was 10 μl. Wavelength of DAD detector was set at 254 nm and flow rate set at 0.30 mL/min. Q-TOF MS analysis was carried out on Triple TOFTM 5600+ high resolution mass spectrometry equipped with ESI source. Data of sample and standards were acquired in negative ion mode. Detection conditions were set at ion spray voltage of - 4500 V, temperature of 550 °C, declustering potential of - 50 V and collision energy of - 28 V. TOF-MS range was at m/z 100-1500 Da and product ions mass range was at m/z 50-1200 Da. Nitrogen was used as nebulizer gas; heater and curtain gas were set at 50, 60 and 30 units. The UHPLC–DAD/Q-TOF MS data were analyzed using PeakViewTM software (Sciex) and identification of compounds was aided by publicly available dataset Chemspider (http://www.chemspider.com/).

### In vivo tumorigenesis study

Athymic nude mice were obtained from Shanghai Laboratory Animal Center (Shanghai, China). Luciferase-expressing H1299 cells suspended in serum-free DMEM were mixed with Matrigel in 1:1 ratio and the mixture was injected into the flank of nude mice. After 1 week, mice were randomly separated into 4 groups and each of these groups respectively received vehicle, ISL (25 mg/Kg), THC (25 mg/Kg) or AZD0530 (20 mg/Kg) intraperitoneally once every 3 days for 5 weeks. Tumor development was monitored by examining fluorescence in Xenogen IVIS-200 In Vivo Imaging System once every3 days as previously described [32, 35]. At the end of treatment, tumors and lungs were collected and fixed with 4% paraformaldehyde. Tumors were also weighed prior to fixation. All procedures were conducted with animal welfare considerations and approved by the Ethical Committee of Shanghai University of Traditional Chinese Medicine.

### Immunohistochemistry

Tumors were collected immediately when mice were euthanized. Paraffin-embedded tissues were sectioned and subjected to IHC to detect Tyr421-phosphorylated cortactin and Tyr925-phosphorylated FAK using the respective antibodies as previously described [32].

### Statistical analysis

Statistical analyses of in vitro invasion, cell migration, tumor development and lung metastasis were performed by ANOVA, using SPSS (release 15.0; SPSS Inc). *p* < 0.05 was considered to be significant.

## Results

### ISL effectively inhibits NSCLC cell migration

To investigate the potency of components in licorice to suppress cell migration, we first performed Transwell and wound-healing assays to evaluate migratory potential on a panel of NSCLC cell lines and observed that H1299, A549, H1975 and H2228 cells were highly migratory while others were either little or poorly migratory (Additional file 1: Figure S1A and B). We next assayed the effect of various licorice constituents including triterpenoids (18-α-glycyrhetinic acid, 18-β-glycyrhetinic acid, glycyrrhizin, enoxocone) and flavonoids (liquiritin, glabridin, carbenoxolone, liquiritigenin, licochalcone A, ISL) on H1299 cell growth. MTT assay revealed that none of these compounds at concentration less than 10 μM significantly affected cell growth in a 2-day treatment period (Additional file 1: Figure S2).

**Fig. 1:**
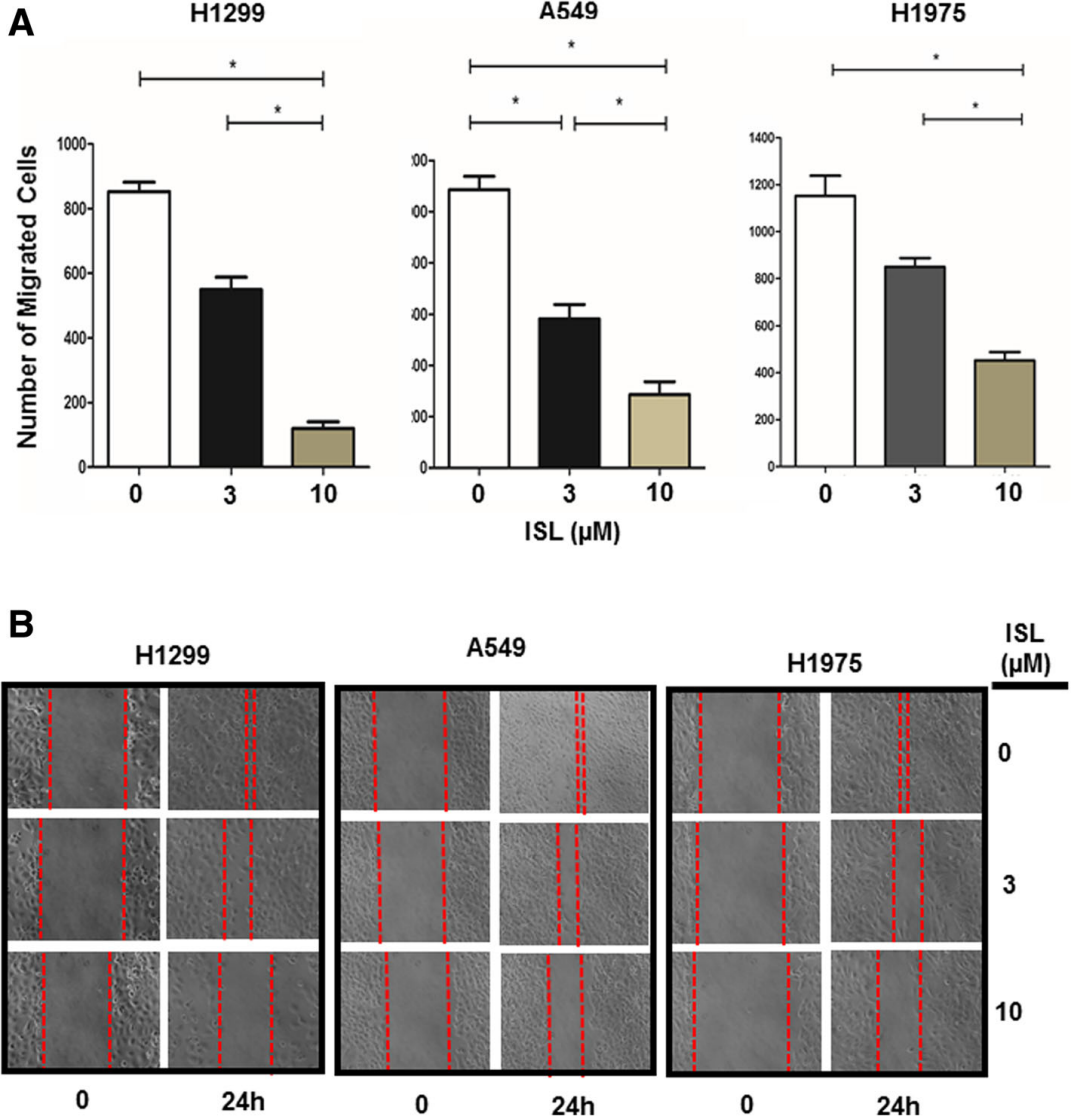
ISL inhibits lung cancer cell migration. **a** Cells were treated with vehicle, 3 or 10 μM ISL for 24 h followed by the analysis of cell migration using Transwells. Data are means ± SD. *n* = 4. *, *p* < 0.01. **b** Cells were grown to confluent followed by treatment of vehicle, 3 or 10 μM ISL for 24 h. A scratch was made with a fine pipette tip and cells were kept in medium containing 1% FCS with or without ISL. Images were taken at 0 and 24 h under a phase contrast microscope

To identify the most effective migration-inhibitory compound in these licorice components, H1299 cells were treated with each of these compounds at 10 μM for 24 h followed by wound-healing assay. Among them, ISL was the only component effectively deterring H1299 cell migration at 10 μM (Additional file 1: Figure S3). To substantiate it, we further determined the effect of ISL on migration of A549, H1299 and H1975 cells at 3 and 10 μM. Transwell and wound-healing assays showed that migration of all 3 lines was dose-dependently inhibited by ISL (Fig. 1a and b). Since ISL up to 30 μM displayed little effect on growth in these lines (Additional file 1: Figure S4), these results demonstrate ISL as an effective compound to suppress lung cancer cell migration.

### ISL deters actin-mediated cytoskeleton reorganization and focal adhesion assembly

**Fig. 2:**
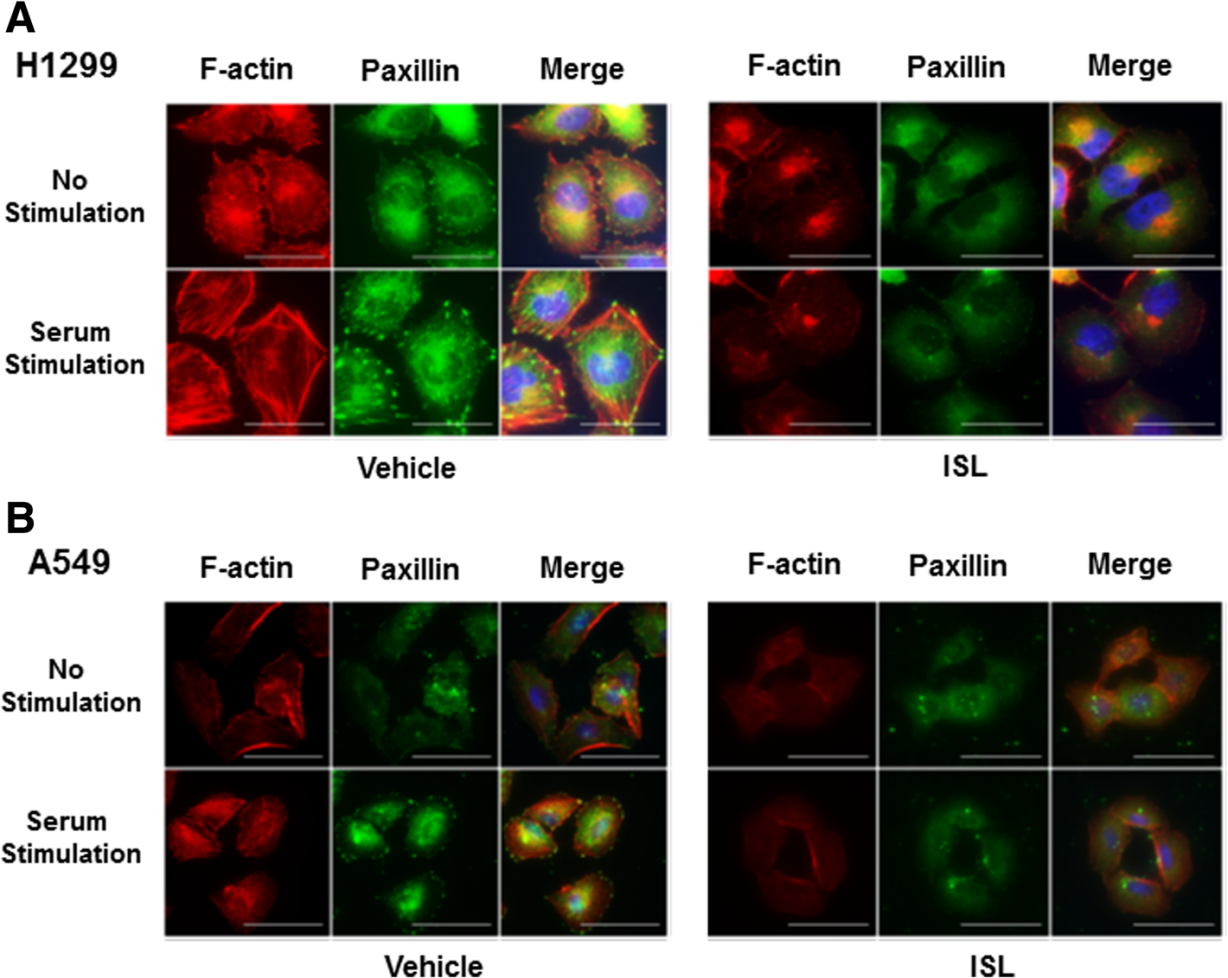
ISL abolishes serum-induced actin reorganization and focal adhesion assembly. H1299 (**a**) and A549 cells (**b**) were treated with vehicle or 10 μM ISL for 24 h in serum-free condition, then stimulated with 10% FCS for 1 h followed by immunostaining with phalloidin (red) and paxillin mAb (green). A representative view was chosen for photography

Cell migration requires the coordinated regulation of actin-mediated cytoskeleton reorganization and focal adhesion assembly [36]. We investigated the effect of ISL on these two events in H1299 and A549 cells. Cells were treated with 10 μM ISL or left untreated for 24 h in serum-free condition and then induced with 10% FCS for 1 h followed by immunostaining with FITC-labeled paxillin antibody and rhodomine-labeled phalloidin. In vehicle-treated cells, we observed weak F-actin staining (phalloidin-staining) in the inner surface of the plasma membrane and a few paxillin-containing focal adhesions (Fig. 2a and b). Serum stimulation elicited not only dramatic actin reorganization (evidenced by strong F-actin staining and the formation of stress fiber crossing the cell bodies) but also significant increase in both the number and size of the focal adhesions (Fig. 2a and b). However, treatment of ISL nearly completely blocked these serum-induced changes (Fig. 2a and b). These results suggest that ISL inhibits cell migration by blocking cytoskeleton reorganization and focal adhesion assembly.

### ISL interferes with cellular Src activity but is not a direct Src inhibitor

**Fig. 3:**
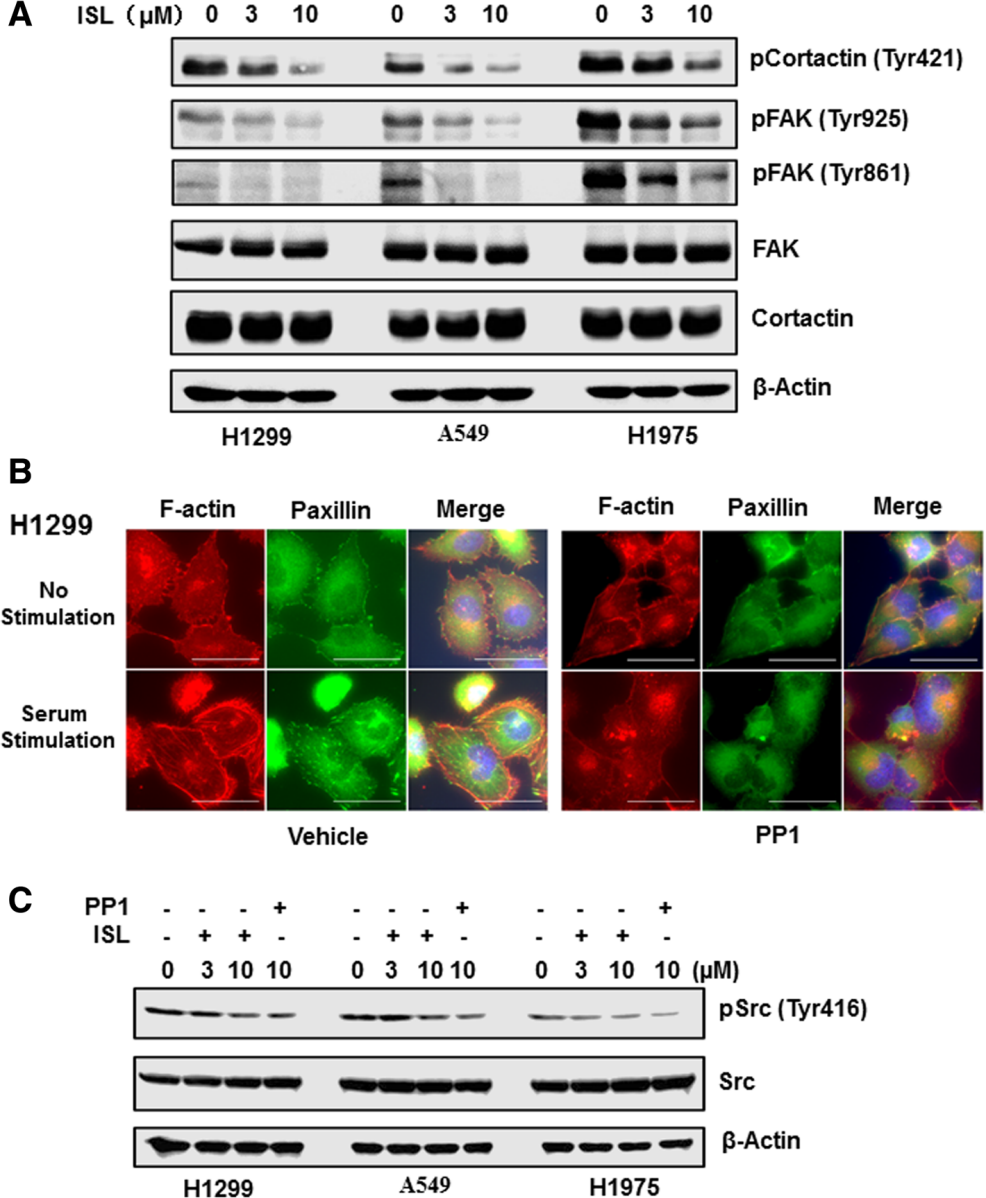
ISL interferes with Src signaling in lung cancer cells. **a** Cells were treated with vehicle, 3 or 10 μM ISL for 24 h and then lysed for western blotting to detect Tyr421-phosphorylated cortactin, Tyr861-, Tyr925-phosphorylated FAK, cortactin, FAK and β-actin with the respective antibodies. **b** H1299 cells were treated with vehicle or 10 μM PP1 for 24 h in serum-free condition, then stimulated with 10% FCS for 1 h followed by immunostaining with phalloidin (red) and paxillin mAb (green). A representative view was chosen for photography. **c** Cells were treated with vehicle, ISL or PP1 for 24 h and then lysed for western blotting to detect Tyr416-phosphorylated Src, Src and β-actin with the respective antibodies

To investigate the mechanism responsible for ISL-led inhibition of cytoskeleton reorganization and focal adhesion assembly, we performed an antibody-based array to compare the phosphorylation status of 141 proteins associated with actin dynamics between untreated and ISL-treated H1299 cells (Additional file 1: Table S2). Among 22 proteins in which their phosphorylation was negatively regulated by ISL, levels of cortactin (Phospho-Tyr421), FAK (Phospho-Tyr925) and FAK (Phopsho-Tyr861) were reduced by more than 30% (Additional file 1: Table S1). To validate these results from array, we treated H1299, A549 and H1975 cells with 3 and 10 μM ISL for 24 h. Western blotting with specific antibodies confirmed that ISL indeed decreased levels of Tyr421-phosphorylated cortactin, Tyr925-phosphorylated and Tyr-861-phosphorylated FAK in both cell lines (Fig. 3a), thus corroborating array results.

Tyr421 of cortactin, Tyr861 and Tyr925 of FAK are known to be the sites phosphorylated by Src. We thus hypothesized that ISL might interfere with Src function. To test this hypothesis, we first treated H1299 and A549 cells with specific Src inhibitor PP1 overnight followed by serum stimulation for 1 h. Immunofluorescence staining showed that, similar to ISL, PP1 blocked serum-induced cytoskeleton reorganization and focal adhesion assembly (Fig. 3b and Additional file 1: Figure S5). Also consistent with our hypothesis, ISL reduced the abundance of Tyr-phosphorylated Src in a similar extent of PP1 (Fig. 3c). To investigate whether ISL inhibits Src activity directly, we analyzed the effect of ISL on the ability of Src kinase to phosphorylate its substrate in cell-free system. While PP1 effectively blocked Src activity with an IC of 379 nM, ISL up to 100 μM displayed no inhibitory effect on Src activity (Additional file 1: Figure S6). These results suggest that ISL interferes with Src function in an indirect mechanism.

### ISL is metabolized to 2, 4, 2′, 4’-Tetrahydroxychalcone which is a direct Src inhibitor

**Fig. 4:**
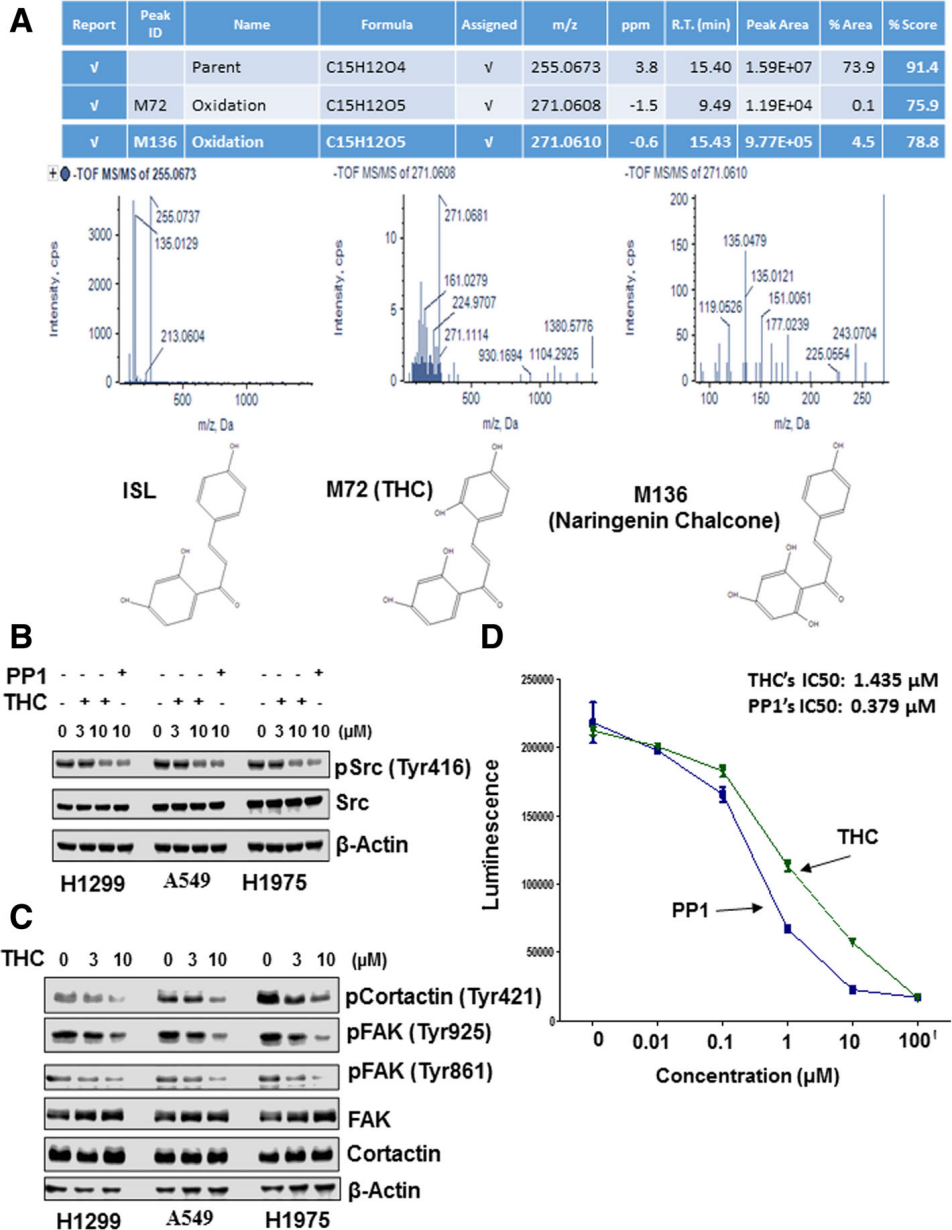
THC is a main ISL metabolite and a Src inhibitor. **a**. Mass spectrum identifies THC and naringenin chalcone as main metabolites of ISL in H1299. **b** Cells were treated with vehicle, THC or PP1 for 24 h and then lysed for western blotting to detect Tyr416-phosphorylated Src, Src and β-actin with the respective antibodies. **c** Cells were treated with vehicle or THC for 24 h and then lysed for western blotting to detect Tyr421-phosphorylated cortactin, Tyr861-, Tyr925-phosphorylated FAK, cortactin, FAK and β-actin with the respective antibodies. **d** Cell-free system to determine the effect of THC and PP1 on Src kinase activity. Data are means ± SD. *n* = 3

The observation that ISL reduced Src phosphorylation in lung cancer cells but was ineffective to inhibit Src activity in cell-free system prompted us to hypothesize that ISL interfered with Src signaling pathway through its metabolites. To test this hypothesis, we performed liquid chromatography-mass spectrometry (LC-MS) on lysates of H1299 cells treated with 10 μM ISL for 24 h and detected two major metabolites of ISL: THC and Naringenin chalcone (Fig. 4a). To test the effect of these two ISL metabolites on Src signaling pathway, we treated H1299, A549 and H1975 cells with these two compounds for 24 h followed by immunoblotting to detect Tyr416-phosphorylated Src. THC, but not Naringenin chalcone reduced the degree of Tyr416-phosphorylated Src in both cell lines (Fig. 4b and Additional file 1: Figure S7). Similar to ISL, THC also reduced the levels of Tyr421-phosphorylated cortactin, Tyr861- and Tyr925-phosphorylated FAK (Fig. 4c). Distinct from ISL, THC was able to inhibit Src kinase activity with IC50 of 1.435 μM in cell-free system (Fig. 4d). Taken together, these results suggest that ISL interferes with Src signaling pathway through its metabolite THC in lung cancer cells.

### THC effectively blocks cell migration and cytoskeleton reorganization

**Fig. 5:**
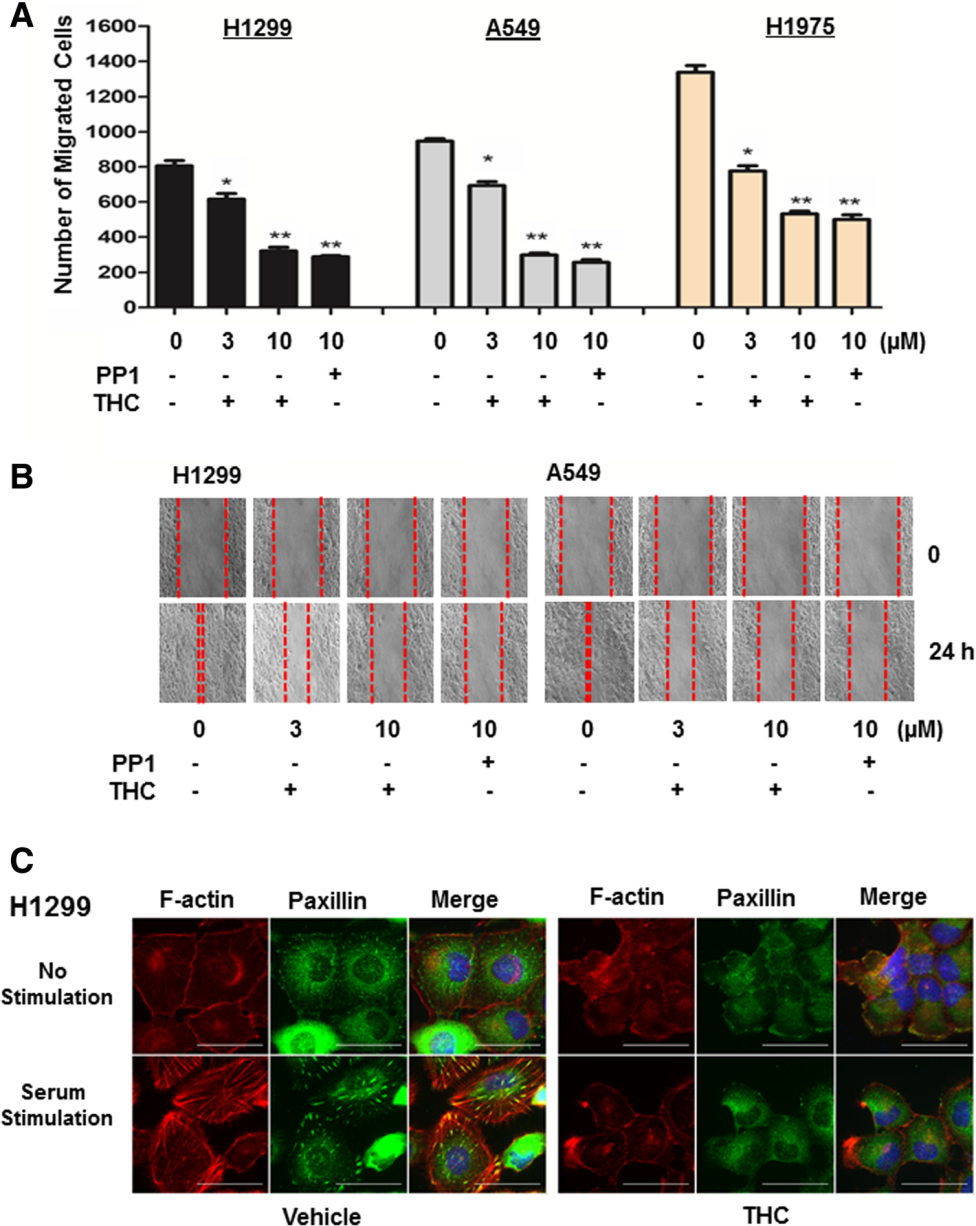
THC deters cell migration and abolishes serum-induced actin reorganization/focal adhesion assembly. **a** Cells were treated with vehicle, THC or PP1 for 24 h followed by the analysis of cell migration using Transwells. Data are means ± SD. *n* = 4. *, *p* < 0.01 vs control (0). **, *p* < 0.005 vs control (0). **b** Cells were grown to confluency followed by treatment of vehicle, THC or PP1 for 24 h. A scratch was made with a fine pipette tip and cells were kept in medium containing 1% FCS with or without THC or PP1. Images were taken at 0 and 24 h under a phase contrast microscope. **c**. H1299 cells were treated with vehicle or 10 μM THC for 24 h in serum-free condition, then stimulated with 10% FCS for 1 h followed by immunostaining with phalloidin (red) and paxillin mAb (green). A representative view was chosen for photography

The identification of THC as a direct Src inhibitor prompted us to examine its effectiveness to suppress cell migration. H1299, H1975 and A549 cells were treated with varying concentration of THC or 10 μM PP1 for 24 h followed by performing Transwell and wound-healing assays to assess cell migration. Similar to what was observed with ISL (Fig. 1a and b), THC dose-dependently inhibited migration of all three cell lines (Fig. 5a and b and Additional file 1: Figure S8). Also similar to ISL, THC at 3 and 10 μM displayed little effect on growth of both A549 and H1299 cells (Additional file 1: Figure S9). At 10 μM, THC reached similar degree of inhibition in cell migration as 10 μM PP1 did (Fig. 5a and b). In a parallel experiment, we analyzed the effect of THC on serum-induced cytoskeleton reorganization and focal adhesion assembly in H1299 and A549 cells. Immunofluorescence staining showed that THC diminished serum-induced stress fiber formation and focal adhesions assembly in both cell lines (Fig. 5c and Additional file 1: Figure S10). These results support the notion that ISL deters cell migration through its metabolite THC, which in turn acts to abolish Src-dependent cytoskeleton reorganization and focal adhesion assembly.

### THC and ISL suppress invasion and metastasis of lung cancer cells

**Fig. 6:**
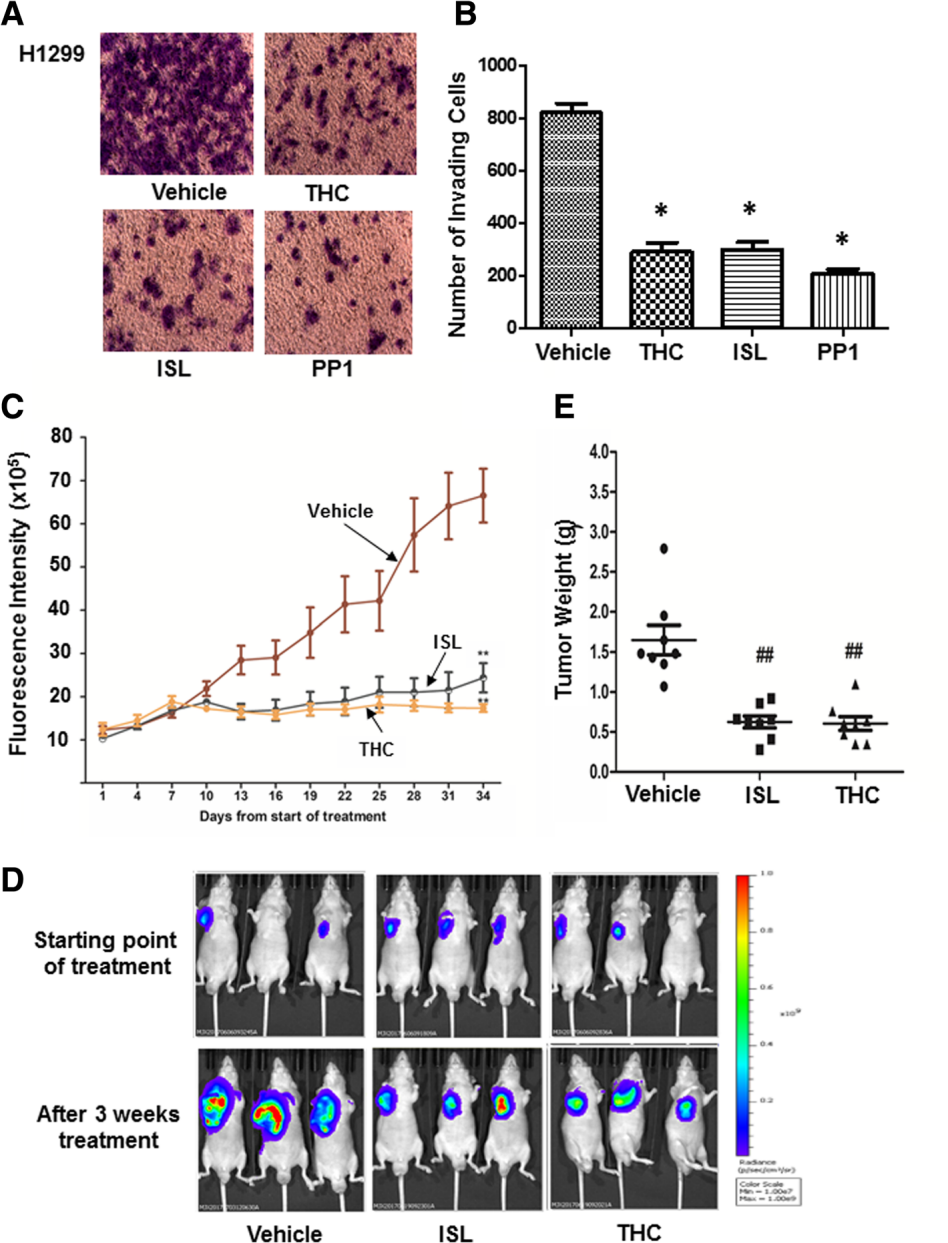
ISL and THC suppress in vitro invasion and in vivo tumor development. **a** H1299 cells were treated with vehicle, 10 μM THC, ISL or PP1 followed by Matrigel invasion assay. Cells on undersurface of invasion chambers were stained and images were taken under a phase contrast microscope. **b** Quantitation of invasive cells (on undersurface). Data are the mean ± SE. *n* = 3. *, *p* < 0.01 vs vehicle. **c** Luciferase-containing H1299 cells were injected into nude mice for 2 weeks followed by administration of 25 mg/Kg ISL or THC. Tumor development was monitored by measuring fluorescence intensity once every 3 days starting from 1 week after tumor cell injection. Data are the mean ± SE. *n* = 8. **d** Tumors were imaged in Xenogen system 3 weeks after treatment. **e** Tumors were excised at the end of treatment and weighed. ##, *p* < 0.05 vs vehicle

The usefulness of Src inhibitors has been demonstrated in various experimental models including lung cancer. The ability of THC and ISL to potently interfere with Src signaling pathways led us to determine their effect on in vitro invasion of lung cancer cells. H1299, A549 and H1975 cells were treated with 10 μM THC or ISL for 1 day and then assessed for their invasiveness using Matrigel invasion chambers. All three cell lines were robustly invasive; however, treatment of ISL or THC abrogated more than 75% of their in vitro invasion, which was similar to the extent of inhibition elicited by 10 μM PP1 (Fig. 6a and b and Additional file 1: Figure S11). We next employed athymic nude mouse model to investigate if THC and ISL were also capable of suppressing in vivo tumor development. Luciferase-containing H1299 cells were subcutaneously injected into athymic nude mice at left flank for 1 week followed by intraperitoneally administering THC or ISL once every other days for 5 weeks. Judging by the fluorescence intensity, tumors propagated rapidly in mice receiving only vehicle while administrating THC or ISL greatly suppressed tumor outgrowth (Fig. 6c and d). Weighing tumors collected from scarified mice revealed that THC or ISL treatment resulted in over 60% reduction in tumor weight (Fig. 6e).

**Fig. 7:**
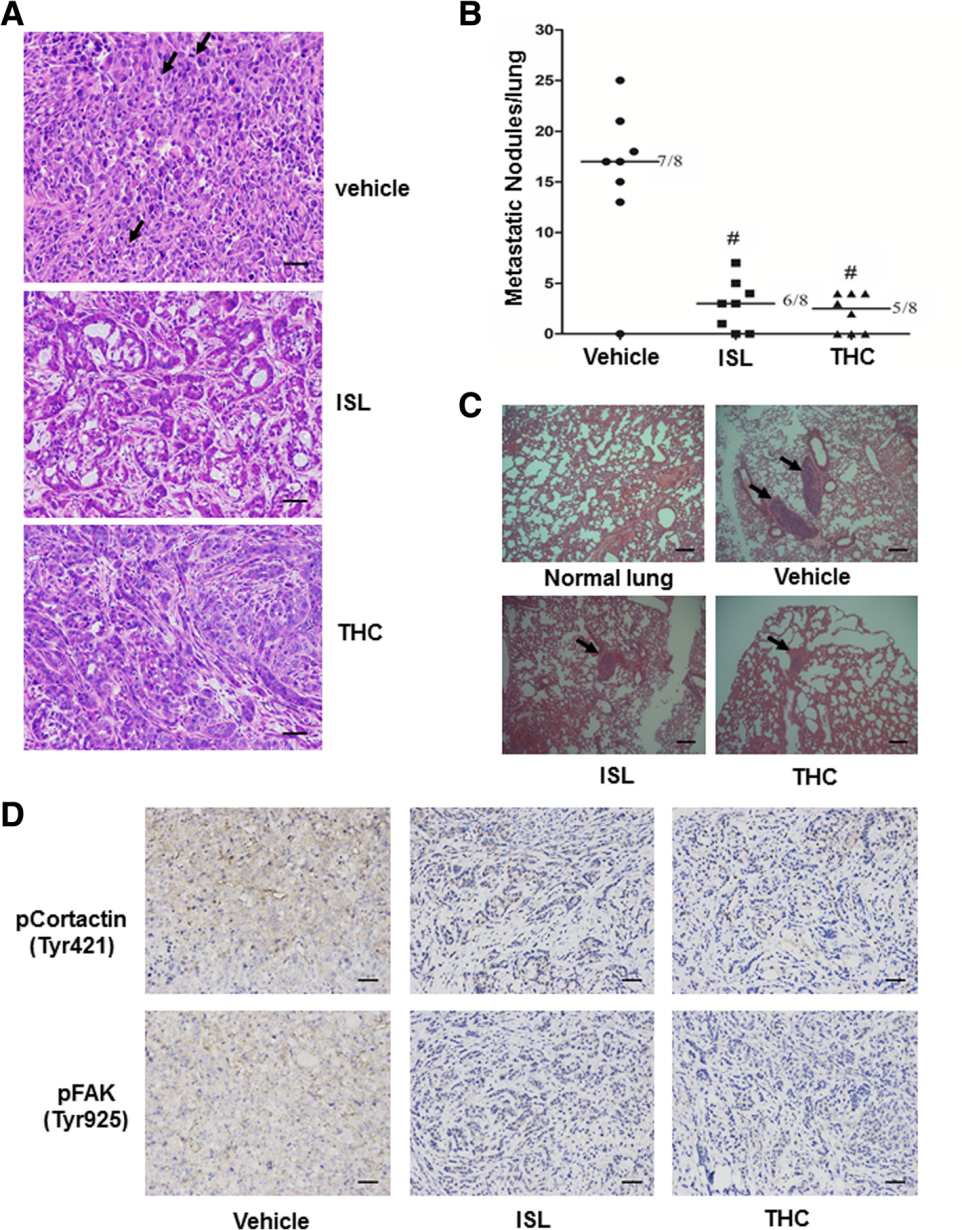
ISL and THC block metastatic potential of lung cancer cells. **a** H&E staining of tumor tissues excised from mice treated with vehicle, ISL or THC. Arrows point at pathological nuclear divisions. Images: 200×; Scale bars: 50 μm. **b** Metastatic nodules in lungs excised from mice treated with vehicle, ISL or THC. #, *p* < 0.001 vs vehicle. **c** Images of H&E staining of lungs excised from mice treated with vehicle, ISL or THC. Arrows point at metastatic nodules. Images: 100×; Scale bars: 200 μm. **d** Representative pictures of immunohistochemical staining of phosphor-Cortactin (Tyr421) and phosphor-FAK (Tyr925) in tumor tissues. Images: 100×; Scale bars: 200 μm

To determine the effect of ISL and THC on metastatic behaviors of H1299 cells, we first performed hematoxylin and eosin (H&E) histological staining on excised tumors. Microscopic analyses of tumor sections showed that tumor cells in vehicle-receiving mice were poorly differentiated, densely distributed and ratio of nucleus to cytoplasm in tumor cells was enhanced (Fig. 7a). Moreover, the nuclei of tumor cells often displayed irregular shapes and there was appearance of increased pathological nuclear divisions (shown by dark arrows, Fig. 7a). In contrast, tumor cells in ISL or THC-treated mice were in an increased differentiation status and displayed nearly uniformed round shape of nuclei (Fig. 7a). In addition, apparent cell death was clearly visible in ISL and THC groups (Fig. 7a). Further microscopic analyses of lung sections revealed an average of 17 metastatic lesions in lungs of vehicle-receiving mice (Fig. 7b). Treatment of ISL or THC not only decreased number of metastatic lesions in lungs but also reduced the size of the lesions (Fig. 7b and c). To link suppressed tumor outgrowth and metastasis to blocked Src signaling pathway, we performed immunohistochemistry to examine the intensity of Tyr421-phosphorylated cortactin and Tyr925-phosphorylated FAK staining on collected tumors. While staining of phosphor-cortactin and phosphor-FAK was strong in tumors derived from vehicle-receiving mice, it was much less in tumors from THC or ISL-treated mice (Fig. 7d).

In parallel, we also investigated the effect of AZD0530, a clinically used Src inhibitor on lung tumor progression. Mice receiving H1299 cells for 1 week were intraperitoneally administered with AZD0530 once every other days for 5 weeks and tumor outgrowth was determined by measuring fluorescence intensity and weighing excised tumors at the end of treatment. Similar to what was observed with ISL and THC, AZD0530 suppressed tumor outgrowth and reduced tumor weight (Additional file 1: Figure S12A and B). Analyzing lung sections also showed that AZD0530 decreased number of metastatic lesions in lungs and also the size of the lesions (Additional file 1: Figure S12C and D). These results are apparently consistent with the notion that THC and ISL suppress tumor progression and metastasis by interfering with Src signaling pathway.

## Discussion

Licorice has long been used as a medical herb in China while is widely used for culinary purposes in Western countries. Various components isolated from licorice have been shown to exhibit anti-tumorigenic effect. For example, glycyrhetinic acid inhibits growth of melanoma [37], androgen dependent prostate cancer [38] and breast cancer cells [39], whereas glabridin induces apoptosis in human hepatoma cells [40]. With the aid of H1299 lung cancer cell lines, we found that several compounds from licorice (18α-glycyrhetinic acid, liquiritin, licochalcon A and ISL) were able inhibit cell growth at the concentration of 30 μM or higher (Additional file 1: Figure S2). However, ISL was able to deter cell migration at concentration < 10 μM and actually was the only compound capable of effectively doing this (Additional file 1: Figure S3, 1A and B). The observation that ISL more potently inhibited cell migration than cell growth is also in stark contrast from other components of licorice in which they were only effective in cell growth suppression. The capability of ISL to negatively regulate both cell migration and growth clearly supports the notion that ISL is the main biologically active components for licorice’s anti-tumorigenic effects [41, 42].

Coordinated cytoskeleton reorganization and focal adhesion assembly are essential for cell migration [43]. While serum elicited actin reorganization and assembly of paxillin in the focal adhesion plaque (Fig. 2a and b and Additional file 1: Figure S5), treatment of ISL abolished these two serum-induced events in lung cancer cells (Fig. 2a and b and Additional file 1: Figure S5), suggesting that ISL deters cell migration by impairing cytoskeleton reorganization. To define the underlying mechanism associated with ISL action, we analyzed the phosphorylation status of proteins associated with cytoskeleton and identified that Tyr421 phosphorylation of cortactin, Tyr861 and Tyr925 phosphorylation of FAK were most affected by ISL (Additional file 1: Table S1 and Fig. 3a). Since these sites are known to be phosphorylated by Src [17, 19, 28], we speculated that ISL interferes with Src function, leading to the deterrence of cell migration. This notion is supported by our observations that 1) Similar to ISL, Src specific inhibitor PP1 also blocked serum-induced cytoskeleton reorganization and focal adhesion assembly (Fig. 3b and Additional file 1: Figure S5); and 2) ISL diminished Tyr416-phosphorylation of Src in lung cancer cells (Fig. 3c). As Src signaling pathway is well documented for its role in cell migration [25], our study thus links inhibitory role of ISL in cell migration to its capability to impair Src signaling pathway.

Butein, a chalcone structurally similar to ISL (Additional file 1: Figure S11), has been previously shown to inhibit Src activity [44]. Intriguingly, cell-free Src kinase assay showed that ISL was unable to inhibit Src activity (Additional file 1: Figure S6). A previous study identified butein as a metabolite of ISL in liver microsome [45]. This raised the possibility that ISL might block Src activity through its metabolite in lung cancer cells. Although butein was not detected, we identified THC and naringenin chlcone as main ISL metabolites in ISL-treated H1299 cells (Fig. 4a). Importantly, THC blocked Src activity both in cell-free kinase assay and in various cell lines (Fig. 4b and d). Comparison of structures between butein and ISL reveals an additional hydroxyl group on ring II (Additional file 1: Figure S13). Since THC also contains an additional hydroxyl group on ring II while inactive naringenin chalcone does not (Additional file 1: Figure S11), we reason that additional hydroxyl group on ring II may be critical for the ability of THC to act as a Src inhibitor. One observation that has to be noted is that the inhibitory effect of THC for Src phosphorylation in lung cancer cells was in the same degree as that of ISL despite that it is an ISL metabolite (Figs. 3c and 4b). Although we do not truly understand why THC was not more effective than ISL for reducing Src phosphorylation, we speculate that stability of THC in culture medium may be less than ISL, thus affecting its potency. In addition, we cannot rule out the possibility that THC might not get into cells as efficiently as ISL.

ISL has been shown to inhibit prostate cancer cell migration in a mechanism possibly involving in decreased JNK/AP-1 signaling [8]. ISL has also been reported to inhibit breast cancer cell migration through downregulating cyclooxygenase-2 (COX-2) signaling [7]. However, these studies did not identify the direct target(s) of ISL. Here, we demonstrated that ISL deterred cell migration by inhibiting Src activity through its metabolite THC (Fig. 5). Earlier studies revealed that Src mediates RalA- and advanced glycation end product-induced AP1 activation [46, 47]. COX-2-facilitated cell migration was shown to be associated with Src signaling [48]. Taken together, it is most likely that ISL inhibits Src activity, leading to the impairment of AP-1 and COX-2 signaling.

A series of investigations has presented evidences that the increase of Src activity is accompanied with the progression of many cancers [49, 50] and the activation status of Src can be used as an indicator for tumor progression [51]. Because of the importance of Src in tumor progression and metastasis [52], Src inhibitors have been developed and several of them (AZD0530, dasatinib, ponatinib, etc) have even been approved to treat malignancies [53]. Blocking Src activity is apparently effective in suppressing lung tumorigenesis as we observed that AZD0530 effectively deterred both lung tumor outgrowth and metastasis (Additional file 1: Figure S12). Given the capability of ISL and THC to interfere with Src signaling, they are expected to possess anti-tumorigenic effects. In fact, both ISL and THC suppressed tumor outgrowth and lung metastasis of lung cancer cells in athymic nude mice (Figs. 6c–e, 7b and c). As remarkable reduction in the intensities of phosphorylated cortactin and FAK staining was detected in excised tumor specimens from these mice (Fig. 7d), these results clearly support the notion that tumor-suppressive effect of ISL and THC is associated with their ability to inhibit Src activity.

## Conclusion

Licorice has long been safely used for both culinary and medical purposes. Our study suggests that ISL or THC may be potentially considered as safe substitute of Src inhibitors.

## Additional file

Additional file 1: **Table S1.** Antibodies used. **Table S2.** Relative levels of phosphorylated proteins associated with actin dynamics. **Figure S1.** Migration of various lung cancer cell lines. **Figure S2.** Effect of licorice’s components on H1299 cell growth. **Figure S3.** Effect of licorice’s components on H1299 cell migration. **Figure S4.** Effect of ISL on growth of various lung cancer cell lines. **Figure S5.** Effect of ISL on serum-induced cytoskeleton reorganization. Figure S6. Effect of ISL and PP1 on Src kinase activity in a cell-free system. **Figure S7.** Effect of naringenin chalcone (NA) on Src activity (abundance of Tyr416 phosphorylated Src) in lung cancer cells. **Figure S8.** Effect of THC on H1975 cell migration. **Figure S9.** Effect of THC on lung cancer cell growth. **Figure S10.** Effect of THC on serum-induced cytoskeleton reorganization. **Figure S11.** Effect of THC, ISL and PP1 on lung cancer cell *in vitro* invasion. **Figure S12.** Effect of AZD0530 on lun tumor progression. **Figure S13.** Structure of butein, ISL, THC and naringenin chalcone. (PDF 2533 kb)

## Abbreviations

ANOVA: Analysis of variance
AP1: Activator protein 1
COX-2: cyclooxygenase-2
DAPI: 4′, 6-diamidino-2-phenylindole
EGFR: Epidermal growth factor receptor
FAK: Focal adhesion kinase
IHC: Immunohistochemistry
ISL: Isoliquiritigenin
JNK: c-Jun N-terminal kinase
MTT: (3-(4,5-Dimethylthiazol-2-yl)-2,5-Diphenyltetrazolium Bromide)
NSCLC: Non-small cell lung carcinomas
PI3K: Phosphoinositide 3-kinase
SFK: Src family kinase
THC: 2, 4, 2′, 4’-Tetrahydroxychalcone
VEGF: Vascular endothelial growth factor
