# Supplementary material for: Suppression of lung cancer progression by isoliquiritigenin through its metabolite 2, 4, 2’, 4’-Tetrahydroxychalcone
Source: J Exp Clin Cancer Res. 2018 Oct 3;37:243. doi: 10.1186/s13046-018-0902-4 (PMC6171243; doi:10.1186/s13046-018-0902-4)
Supplement: Supplementary file 1 — Table S1. Antibodies used. Table S2. Relative levels of phosphorylated proteins associated with actin dynamics. Figure S1. Migration of various lung cancer cell lines. Figure S2. Effect of licorice’s components on H1299 cell growth. Figure S3. Effect of licorice’s components on H1299 cell migration. Figure S4. Effect of ISL on growth of various lung cancer cell lines. Figure S5. Effect of ISL on serum-induced cytoskeleton reorganization. Figure S6. Effect of ISL and PP1 on Src kinase activity in a cell-free system. Figure S7. Effect of naringenin chalcone (NA) on Src activity (abundance of Tyr416 phosphorylated Src) in lung cancer cells. Figure S8. Effect of THC on H1975 cell migration. Figure S9. Effect of THC on lung cancer cell growth. Figure S10. Effect of THC on serum-induced cytoskeleton reorganization. Figure S11. Effect of THC, ISL and PP1 on lung cancer cell in vitro invasion. Figure S12. Effect of AZD0530 on lun tumor progression. Figure S13. Structure of butein, ISL, THC and naringenin chalcone. (PDF 2533 kb) [file 13046_2018_902_MOESM1_ESM.pdf]

Supplemental Materials

Table S1. Antibodies Used

Antibody name	Vendors	Cat #	Titer	Application
Paxillin mAb, clone 5H11	Millipore	05-417	1:100	immunostaining
Rhodamine Phalloidin	ThermoFisher	R415	1:40	immunostaining
Phospho-Cortactin (Tyr421)	Cell Signaling Technology	4569S	1:1000	Western blotting
Cortactin	Cell Signaling Technology	3502S	1:1000	Western blotting
FAK	Cell Signaling Technology	3285S	1:1000	Western blotting
Phospho-FAK (Tyr925)	Cell Signaling Technology	3284S	1:1000	Western blotting
Phospho-FAK (Tyr861)	ThermoFisher	44-626G	1:1000	Western blotting
β-Actin (8H10D10)	Cell Signaling Technology	3700S	1:1000	Western blotting
Src	Cell Signaling Technology	2108S	1:1000	Western blotting
Phospho-Src Family (Tyr416)	Cell Signaling Technology	6943S	1:1000	Western blotting
Phospho-FAK(Tyr925)	Bioworld	BS7418	1:50	IHC
Cortactin (phospho-Tyr421)	Bioworld	BS4778	1:50	IHC

Table S2. Relative Levels of Phosphorylated Proteins Associated with Actin Dynamics

Name	ISL/vehicle
Cortactin (Phospho-Tyr421)	0.62
FAK (Phospho-Tyr925)	0.63
FAK (Phospho-Tyr861)	0.66
CaMK2 (Phospho-Thr286)	0.73
Ezrin (Phospho-Tyr353)	0.77
VASP (Phospho-Ser238)	0.79
Calmodulin (Phospho-Thr79/Ser81)	0.81
ERK1-p44/42 MAP Kinase (Phospho-Tyr204)	0.82
MEK1 (Phospho-Ser221)	0.83
MKK3 (Phospho-Ser189)	0.84
Paxillin (Phospho-Tyr31)	0.84
c-Raf (Phospho-Ser43)	0.85
Ezrin (Phospho-Tyr478)	0.85
Filamin A (Phospho-Ser2152)	0.88
cofilin (Phospho-Ser3)	0.88
ERK1-p44/42 MAP Kinase (Phospho-Thr202)	0.89
ERK3 (Phospho-Ser189)	0.89
FAK (Phospho-Ser910)	0.9
CrkII (Phospho-Tyr221)	0.9
Paxillin (Phospho-Tyr118)	0.9
p130Cas (Phospho-Tyr165)	0.9

Supplemental Figure 1

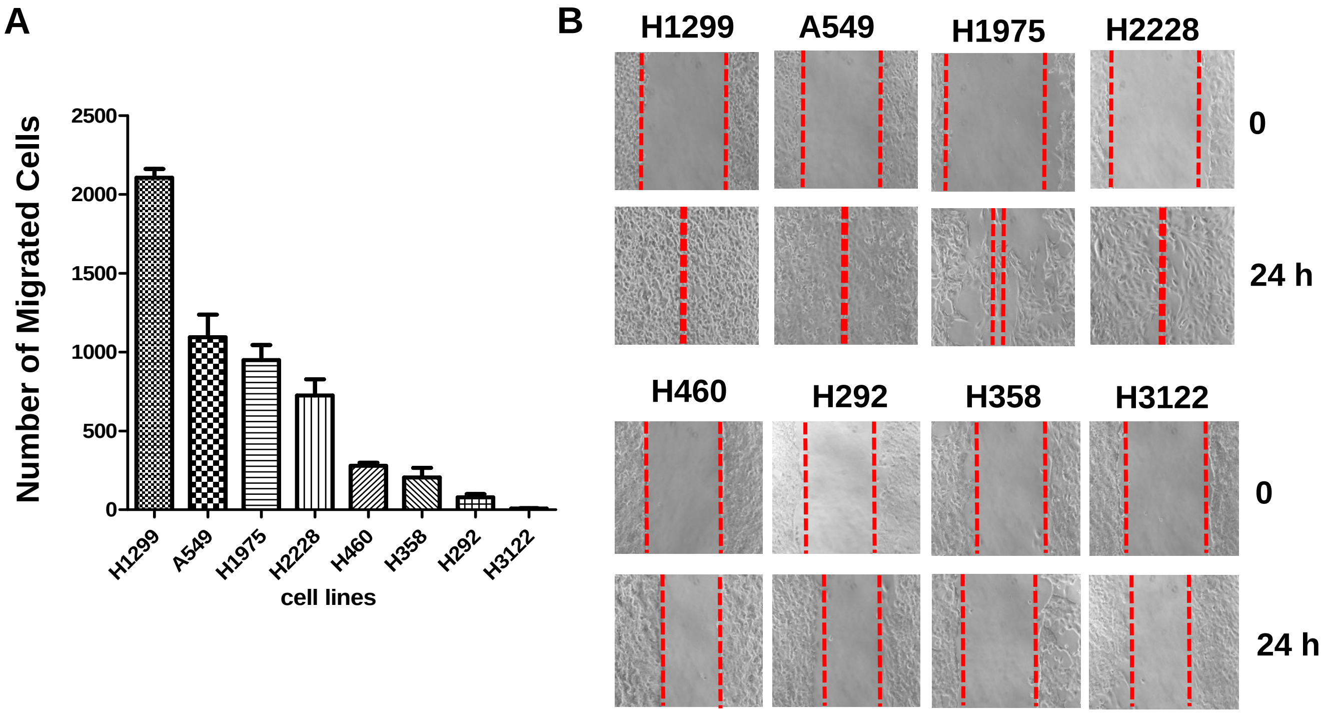

Figure S1. Migration of various lung cancer cell lines. ***A***. Transwell assay. Cells were added into upper chamber of Transwells and allowed to migrate for 4 h. Cells remaining in the upper chamber were removed with cotton swab and cells on undersurface were stained with crystal violet. Stained cells were counted under a phase-contrast microscope. ***B***. Cells were grown to confluency and a scratch was made with a thin pipet tip. Dislodged cells were washed away and cells were left in cell culture incubator for 24 h. Pictures were taken at 0 and 24 h of incubation.

Supplemental Figure 2

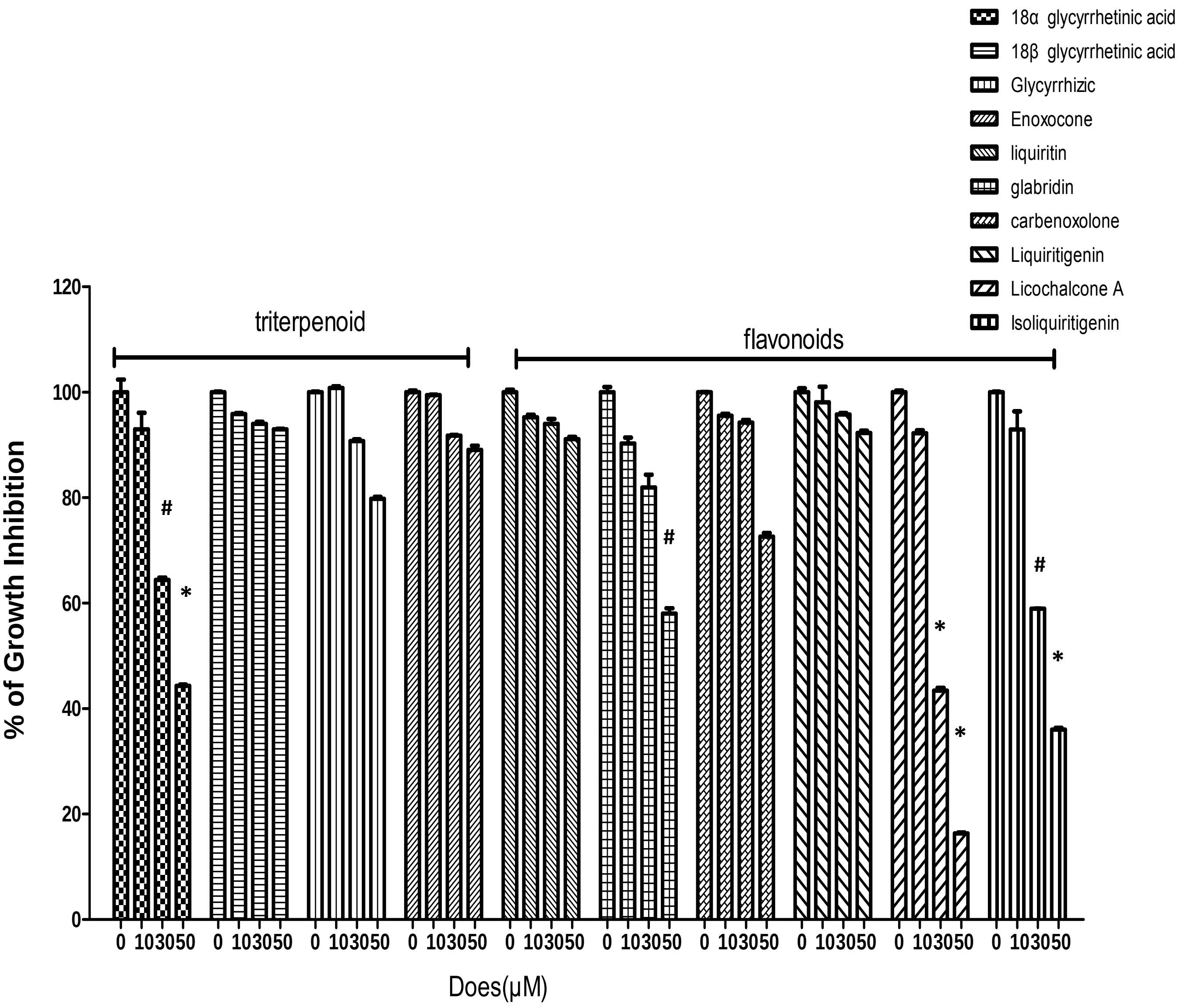

Figure S2. Effect of licorice's components on H1299 cell growth. H1299 cells (5,000 cells/well) were seeded into 24-well plate overnight and various concentration of licorice's components were added to cells for 2 days. MTT was performed to measure cell numbers with the aid of a microplate reader. % of Growth Inhibition was calculated by the formula of $[1\text{-}OD_{drug}/OD_{vehicle}]$x100. Data are mean ± SD; n = 4. *, $p < 0.01$ *vs* vehicle (0). #, $p < 0.05$ *vs* vehicle (0).

Supplemental Figure 3

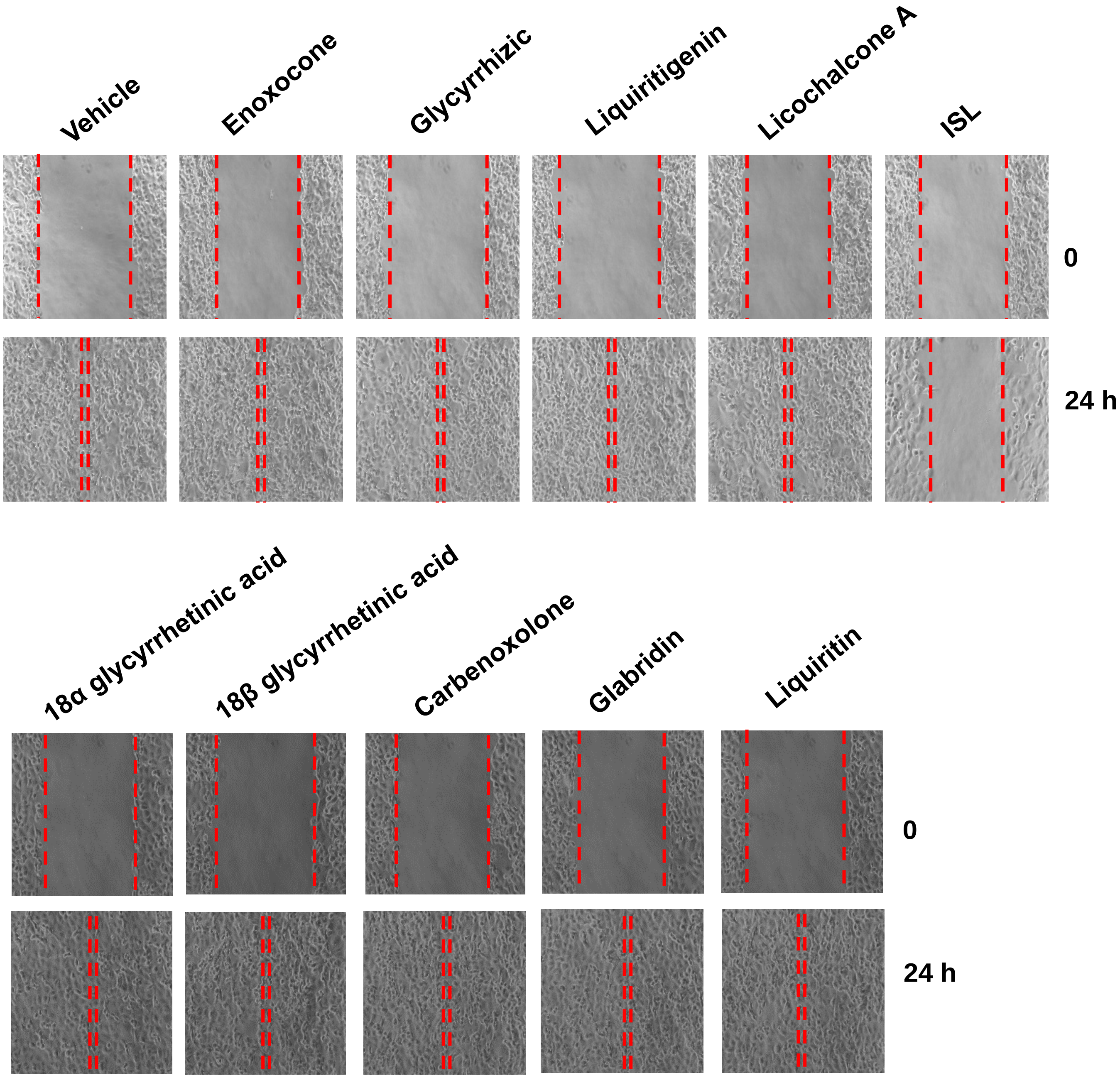

Figure S3. Effect of licorice's components on H1299 cell migration. H1299 cells were grown to confluency and then treated with 10μM of each Licorice components for 24 h before a scratch was made. After removing dislodged cells, cells were left in cell culture incubator for 24 h. Pictures were taken at 0 and 24 h of incubation.

Supplemental Figure 4

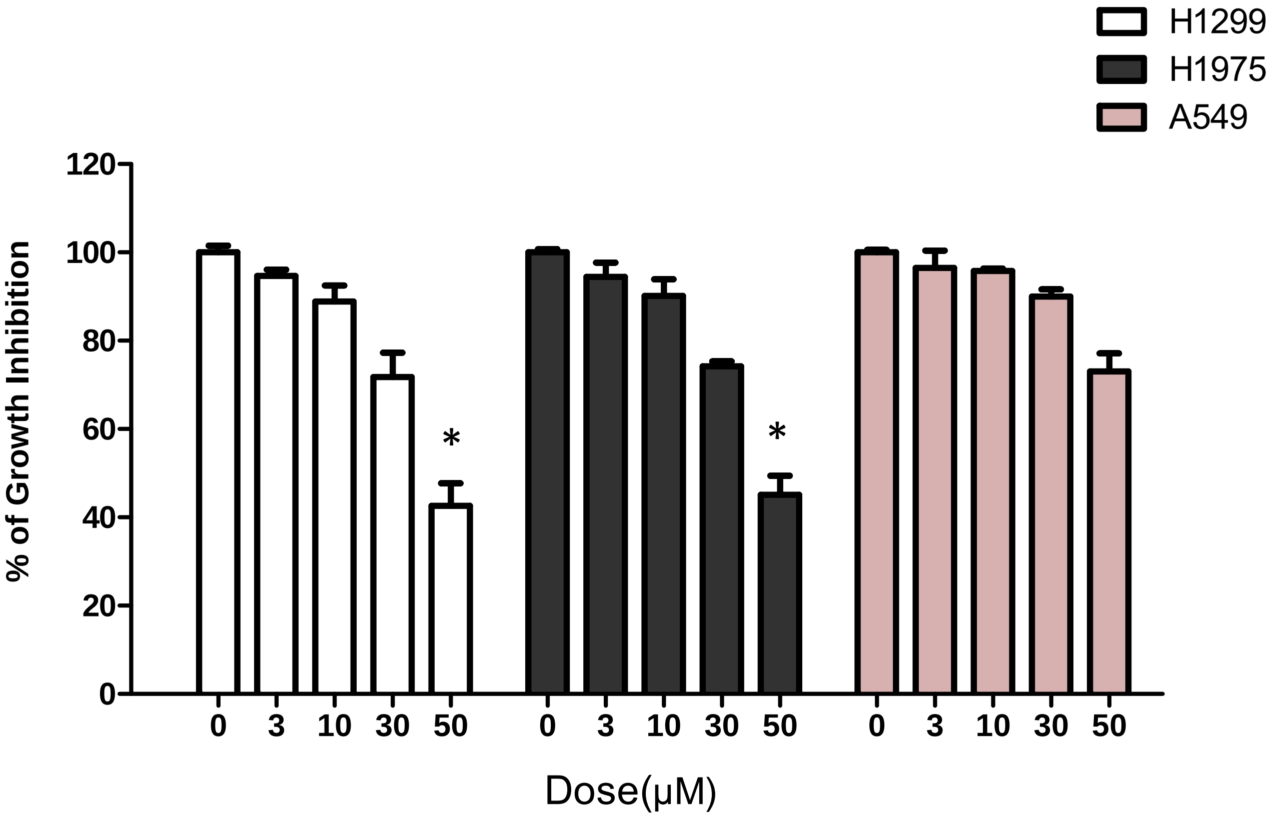

Figure S4. Effect of ISL on growth of various lung cancer cell lines. Cells ($1x10^4$/well in 24-well plate) were treated with varying concentration of ISL for 2 days followed by MTT assay to determine cell growth. % of Growth Inhibition was calculated by the formula of $[1-OD_{drug}/OD_{vehicle}]x100$. Data are mean ± SD; n = 4. *, $p < 0.05$ *vs* vehicle (0).

Supplemental Figure 5

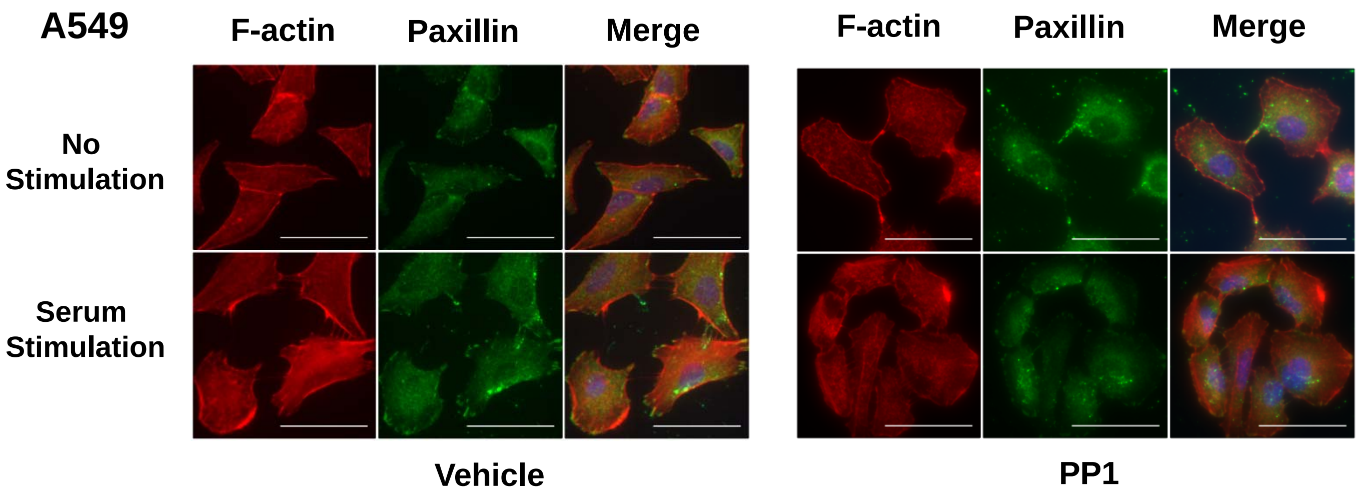

Figure S5. Effect of ISL on serum-induced cytoskeleton reorganization. A549 cells were starved in the absence or presence of 10μM ISL for 24 h followed by serum stimulation for 1 h. Cells were washed, permeabilizaed and then subjected to immunostaining with Rhodamine-labeled phalloidin and FITC-labed paxillin mAb. Cells were visualized under a fluorescence microscope.

Supplemental Figure 6

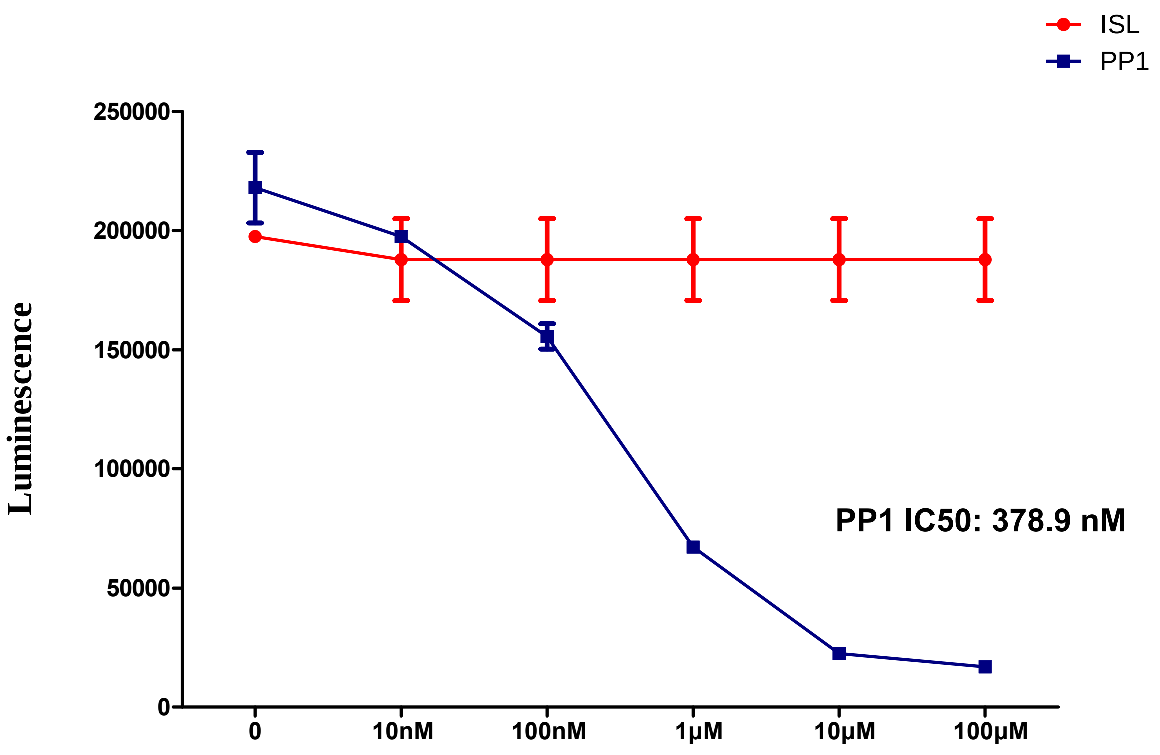

Figure S6. Effect of ISL and PP1 on Src kinase activity in a cell-free system. Src activity was analyzed using ADP-GloTM Src kinase assay kit. Varying concentration of either PP1 or ISL was added into reaction mixture and 30 min reaction period was allowed. Data are mean ± SD; n = 3.

Supplemental Figure 7

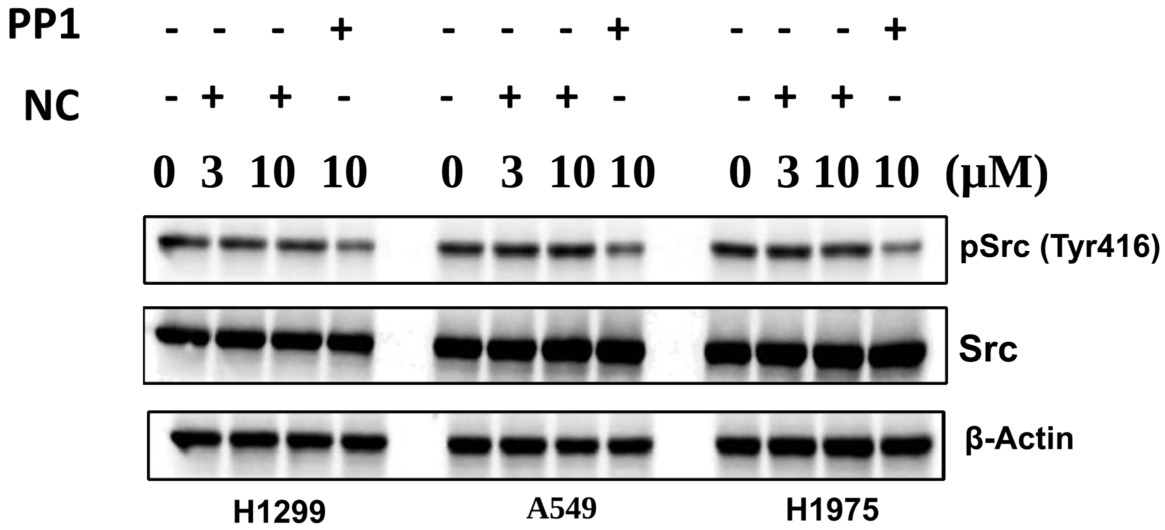

Figure S7. Effect of naringenin chalcone (NA) on Src activity (abundance of Tyr416 phosphorylated Src) in lung cancer cells. Cells were treated with NC (3 and 10μM), 10μM PP1 or vehicle for 24 h and then lysed for Western blotting to determine the abundance of Tyr416 phosphorylated Src, Src and β-Actin with the respective antibodies.

Supplemental Figure 8

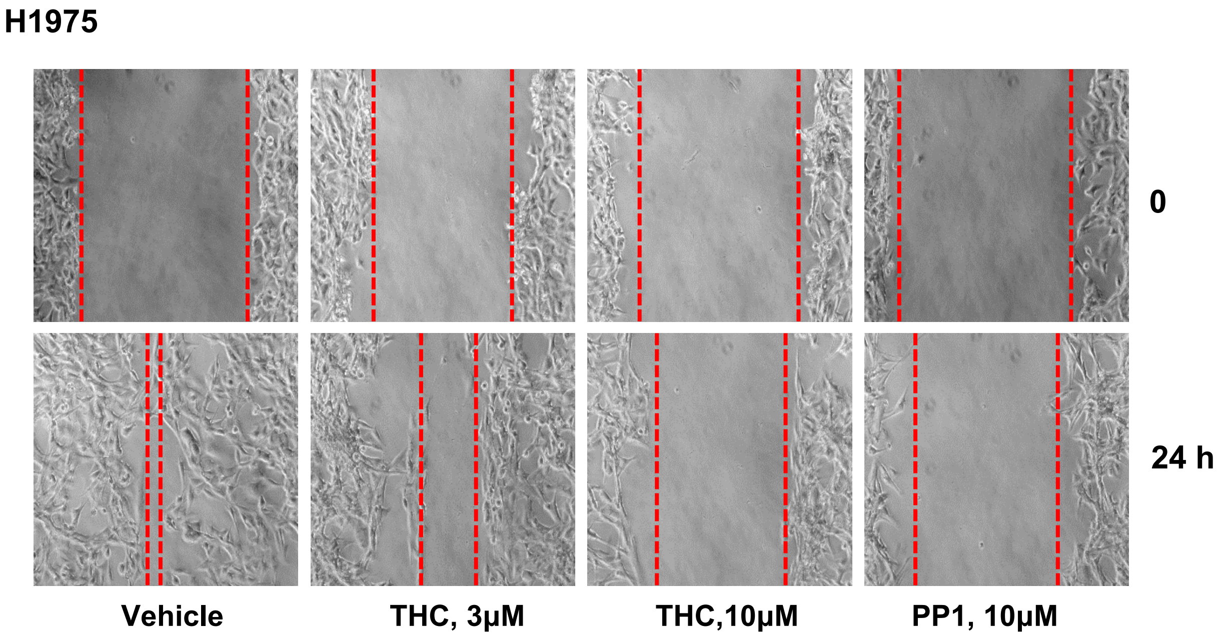

Figure S8. Effect of THC on H1975 cell migration. H1299 cells were grown to confluency and then treated with THC (3 and 10µM), PP1 or vehicle for 24 h before a scratch was made. After removing dislodged cells, cells were left in cell culture incubator for 24 h. Pictures were taken at 0 and 24 h of incubation.

Supplemental Figure 9

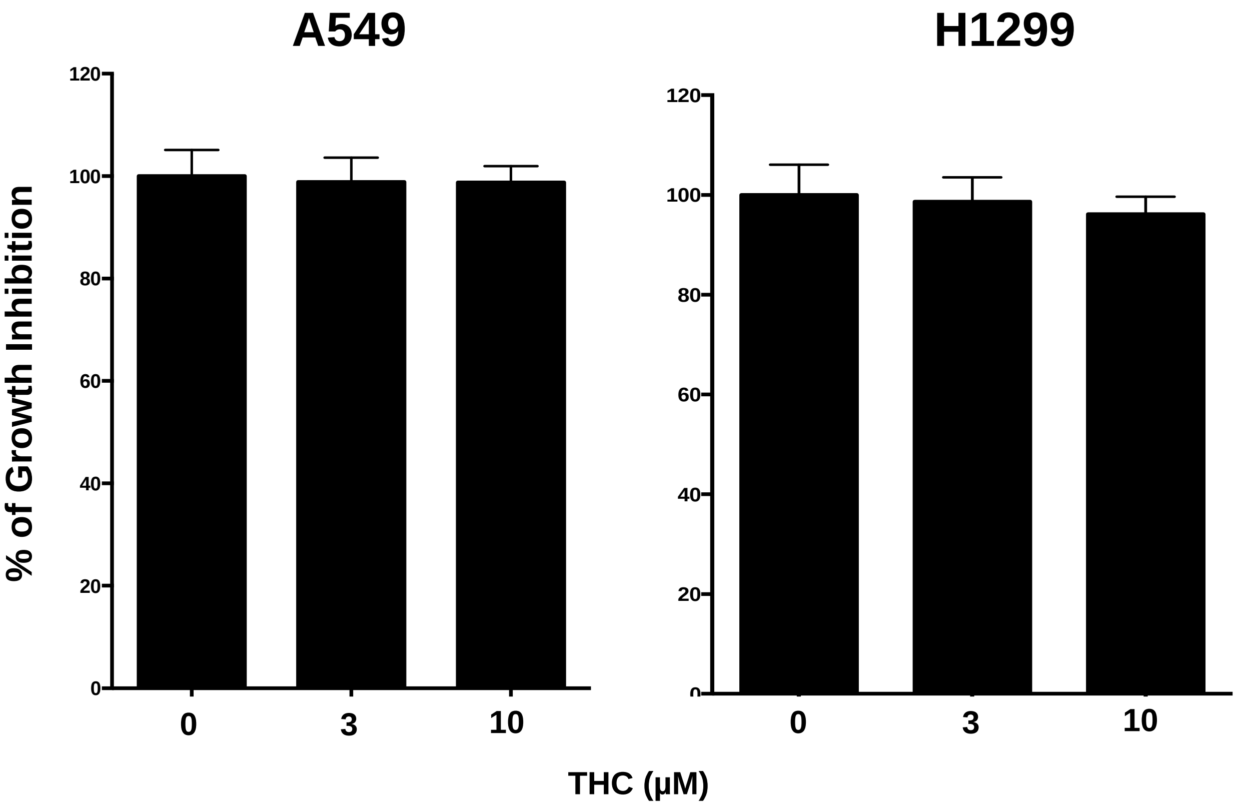

Figure S9. Effect of THC on lung cancer cell growth. A549 and H1299 cells were cultured in the absence or presence of 3 or 10μM THC for 24 h followed by MTT assay to assess cell growth. % of growth inhibition was calculated by the formula $[(OD_0-OD_{THC})/OD_{THC}] \times 100$. Data are means ± SD (n = 4).

Supplemental Figure 10

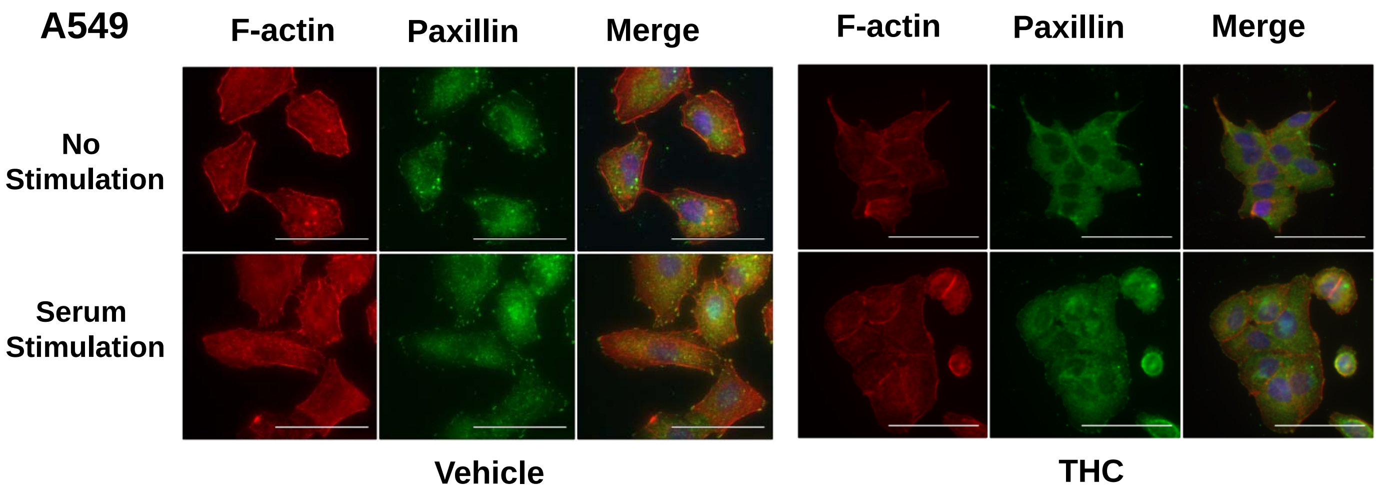

Figure S10. Effect of THC on serum-induced cytoskeleton reorganization. A549 cells were starved in the absence or presence of 10μM ISL for 24 h followed by serum stimulation for 1 h. Cells were washed, permeabilizaed and then subjected to immunostaining with Rhodamine-labeled phalloidin and FITC-labeled paxillin mAb. Cells were visualized under a fluorescence microscope.

Supplemental Figure 11

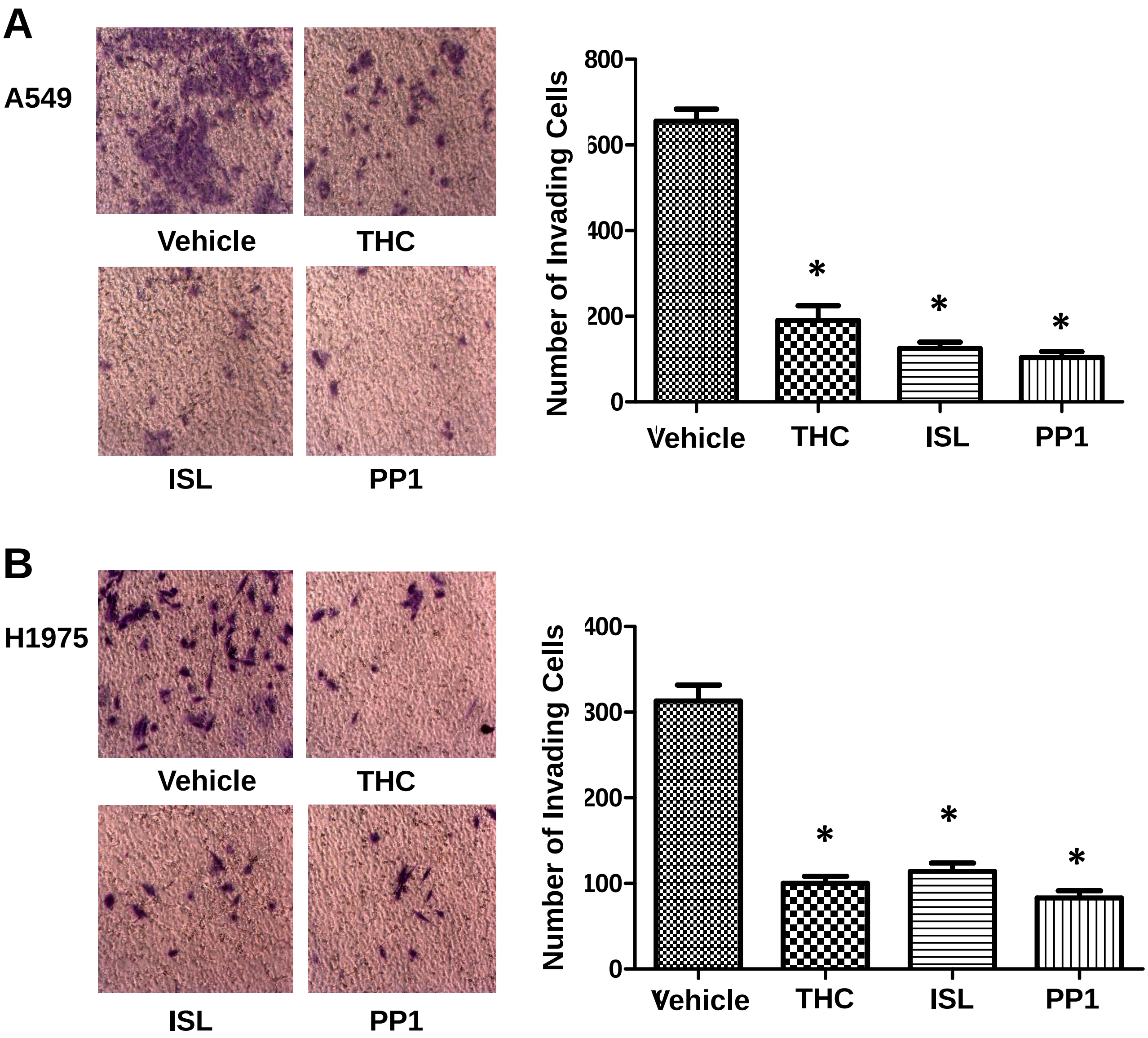

Figure S11. Effect of THC, ISL and PP1 on lung cancer cell *in vitro* invasion. Cells were treated with vehicle, 10µM THC, 10µM ISL or 10µM PP1 for 24 h, then added into invasion chambers ($2x10^5$/chamber) and allowed to invade for 24 h. Cells remaining in the invasion chambers were removed and cells on the undersurface of chambers were stained. Stained cells on the undersurface were counted under a phase-contrast microscope. Data are mean ± SD. n = 3. *, $p < 0.005$ *vs* vehicle.

Supplemental Figure 12

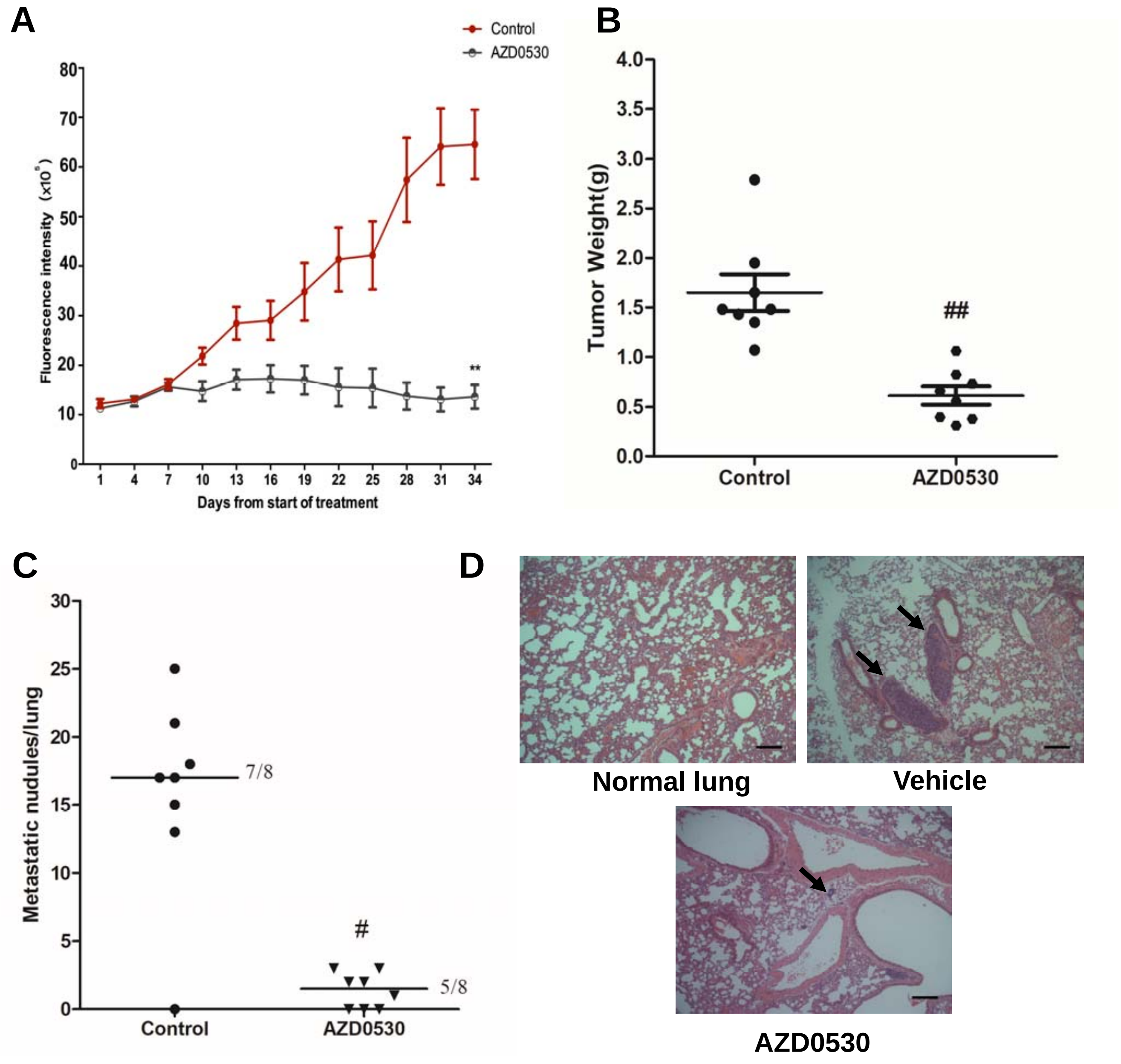

Fig.S12. Effect of AZD0530 on lun tumor progression. ***A***. H1299 cells were injected into nude mice for 2 weeks followed by administration of 20mg/Kg AZD0530 once every other days. Tumor development was monitored by measuring fluorescence intensity one every 3 days starting from 1 week after tumor cell injection. Data are the mean ±SE. n=8. ***B***. Tumors were excised at the end of treatment and weighed. ##, p < 0.05 *vs* vehicle. ***C***. Metastatic nodules in lungs excised from mice treated with vehicle or AZD0530. #, p < 0.001 *vs* vehicle. ***D***. Images of H&E staining of lungs excised from mice treated with vehicle or AZD0530. Arrows point at metastatic nodules. Images: 100X; Scale bars: 200µm.

Supplemental Figure 13

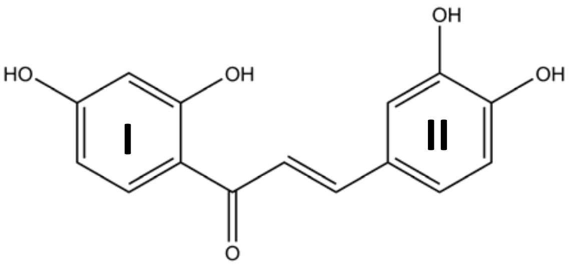

butein

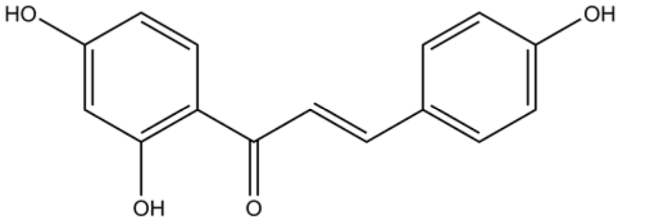

ISL

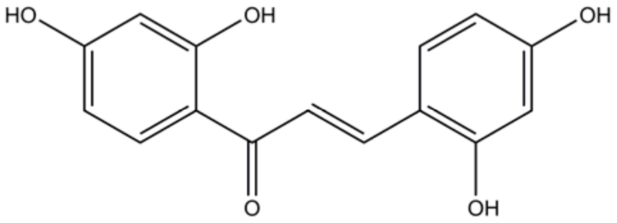

THC

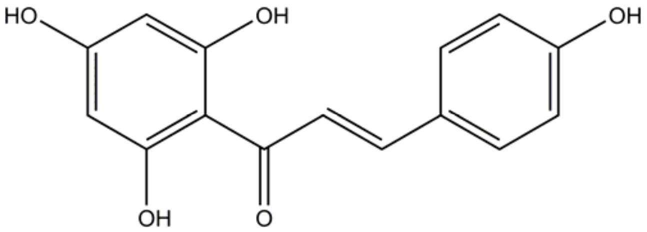

Naringenin chalcone

Figure S13. Structure of butein, ISL, THC and naringenin chalcone.
